# A high-resolution route map reveals distinct stages of chondrocyte dedifferentiation for cartilage regeneration

**DOI:** 10.1038/s41413-022-00209-w

**Published:** 2022-04-27

**Authors:** Yishan Chen, Yeke Yu, Ya Wen, Juan Chen, Junxin Lin, Zixuan Sheng, Wenyan Zhou, Heng Sun, Chengrui An, Jiansong Chen, Weiliang Wu, Chong Teng, Wei Wei, Hongwei Ouyang

**Affiliations:** 1 Dr. Li Dak Sum & Yip Yio Chin Center for Stem Cells and Regenerative Medicine, and Department of Orthopedic Surgery of the Second Affiliated Hospital, Zhejiang University School of Medicine, Hangzhou, 310058 China; 2 Department of Sports Medicine, Zhejiang University School of Medicine, Hangzhou, 310058 China; 3 Zhejiang University-University of Edinburgh Institute, Zhejiang University School of Medicine, and Key Laboratory of Tissue Engineering and Regenerative Medicine of Zhejiang Province, Zhejiang University School of Medicine, Hangzhou, 310058 China; 4 Department of Oral Surgery, Ninth People’s Hospital, Shanghai Jiao Tong University School of Medicine, Shanghai, 200011 China; 5 Department of General Intensive Care Unit, The Second Affiliated Hospital of Zhejiang University School of Medicine, Hangzhou, 310009 China; 6 Department of Orthopedic Surgery, The Children’s Hospital, Zhejiang University School of Medicine, National Clinical Research Center for Child Health, Hangzhou, 310052 China; 7 Department of Orthopedic Surgery, The Fourth Affiliated Hospital, Zhejiang University School of Medicine, Yiwu, 322000 China; 8 China Orthopedic Regenerative Medicine Group (CORMed), Hangzhou, 310058 China

**Keywords:** Diseases, Energy metabolism

## Abstract

Articular cartilage damage is a universal health problem. Despite recent progress, chondrocyte dedifferentiation has severely compromised the clinical outcomes of cell-based cartilage regeneration. Loss-of-function changes are frequently observed in chondrocyte expansion and other pathological conditions, but the characteristics and intermediate molecular mechanisms remain unclear. In this study, we demonstrate a time-lapse atlas of chondrocyte dedifferentiation to provide molecular details and informative biomarkers associated with clinical chondrocyte evaluation. We performed various assays, such as single-cell RNA sequencing (scRNA-seq), live-cell metabolic assays, and assays for transposase-accessible chromatin with high-throughput sequencing (ATAC-seq), to develop a biphasic dedifferentiation model consisting of early and late dedifferentiation stages. Early-stage chondrocytes exhibited a glycolytic phenotype with increased expression of genes involved in metabolism and antioxidation, whereas late-stage chondrocytes exhibited ultrastructural changes involving mitochondrial damage and stress-associated chromatin remodeling. Using the chemical inhibitor BTB06584, we revealed that early and late dedifferentiated chondrocytes possessed distinct recovery potentials from functional phenotype loss. Notably, this two-stage transition was also validated in human chondrocytes. An image-based approach was established for clinical use to efficiently predict chondrocyte plasticity using stage-specific biomarkers. Overall, this study lays a foundation to improve the quality of chondrocytes in clinical use and provides deep insights into chondrocyte dedifferentiation.

## Introduction

Cartilage injury and degradation have become major causes of pain and disability,^1^ affecting over 250 million people worldwide and costing 1%–2.5% of the gross domestic product in developed countries.^2^ Adult articular cartilage shows poor self-renewal, with chondrocytes accounting for only 1%–10% of the whole tissue weight.^3,4^ For cartilage regeneration, cell therapy, such as autologous chondrocyte implantation (ACI),^5,6^ can provide an alternative cell resource with a higher regenerative capacity.

However, the functional phenotype of chondrocyte resources is unstable but critical for cartilage regeneration.^7^ Attaining a workable number of chondrocytes for ACI requires 2–3 weeks of chondrocyte expansion after biopsy,^8^ during which time articular chondrocytes gradually fail to express functional lineage markers and adopt a fibroblast-like morphology with an abnormal extracellular matrix (ECM).^8^ This phenomenon has been reported since 1972^9^ and has been defined as chondrocyte dedifferentiation. Presently, chondrocyte dedifferentiation still hinders the application of cartilage regenerative therapy, as dedifferentiated chondrocytes result in inferior repaired tissues with dysfunctional fibrocartilage in vivo.^10^ Similar phenotype loss during in vitro culture has been reported in other lineages, including hepatocytes and cardiac tissue.^11,12^ Accumulating studies have highlighted the need to dissect and manipulate cellular functional phenotypes, which brings a new perspective to tissue regeneration.^13–15^

Over the past few decades, efforts have been made to monitor the complex process of chondrocyte dedifferentiation. Traditional observations have been made based on cell morphology and simple sets of biomarkers.^16,17^ Studies using microarrays and methylation profiling have reported that chondrocyte dedifferentiation is due to complicated mechanisms,^18–20^ including the mechanical microenvironment,^21,22^ epigenetic factors,^20,23^ oxidative stress^24^ and senescence.^25^ Moreover, the phenotypic alteration of chondrocytes during the dedifferentiation process was not linear over the time course; primary chondrocytes underwent rapid changes upon isolation, but irreversible transformation appeared later at passage 4 (P4).^26–28^ These results indicate a potential biphasic switch during chondrocyte dedifferentiation, but this event has not been confirmed and is not well understood.

Furthermore, the identification of the chondrocyte dedifferentiation process is important in clinical practice. Characterized chondrocyte implantation (CCI) was developed to optimize ACI outcomes, necessitating a precise chondrocyte phenotype evaluation before implantation.^29–31^ Therefore, the development of a high-resolution biomarker atlas that covers the different stages of chondrocyte dedifferentiation would be useful for the assessment of chondrocyte quality and prediction of the regenerative potential of ACI.

Overall, it is extremely difficult to evaluate and manipulate chondrocyte functional phenotypes if the mechanisms underlying chondrocyte dedifferentiation are unclear. Two aspects are still unknown: (1) the molecular events that lead to the irreversible loss of phenotype functionality and (2) a method to objectively identify the different stages of chondrocyte dedifferentiation. To address these issues, in this study, we employed single-cell RNA sequencing (scRNA-seq) of serial-passaged murine chondrocytes (passage 0, 2, 4 and 8) to construct a time-lapse atlas of chondrocyte dedifferentiation. We identified early dedifferentiated chondrocyte subpopulations with transient glycolytic activation and late subpopulations featuring structural changes of metabolic stress in mitochondria and chromatin accessibility. Using a chemical inhibitor that targeted mitochondrial F1Fo-ATPase, we demonstrated the distinct recovery potentials of the early and late stages. Last, the biphasic model was confirmed in human chondrocytes, and dedifferentiation stage indicators defined by scRNA-seq successfully predicted donor chondrocyte quality. Overall, this study provides insights into chondrocyte dedifferentiation, targets for phenotype maintenance, and potential biomarkers for chondrocyte phenotype evaluation.

## Results

### Establishment of a time-lapse chondrocyte dedifferentiation model

To establish a time-lapse chondrocyte dedifferentiation model, we cultured primary hyaline chondrocytes isolated from uncalcified cartilage in knee joints of newborn mice^32,33^ and replated each passage after doubling the cell quantity (seeding density: 2.4 × 10^4^ cells per cm^2^; collecting density: 4.8 × 10^4^ cells per cm^2^). The morphology of cells from passages (P) 0, 2, 4, and 8 was examined. During continuous culture, the chondrocyte morphology typically changed from round/polygonal to flattened/amoeboid-like, and the cellular size increased (Fig. S1A, B).

**Fig. 1:**
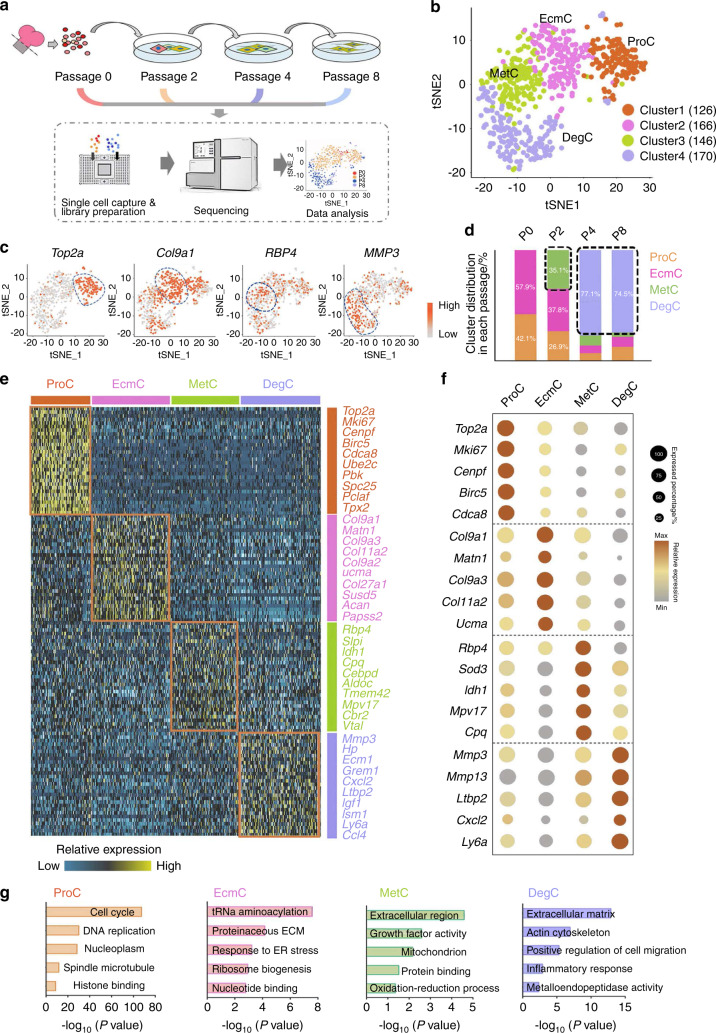
ScRNA-seq identifies distinct cell subpopulations in the early and late passages. **a** The strategy for chondrocyte passaging and the scRNA-seq workflow. **b** T-distributed stochastic neighbor embedding (t-SNE) plotting of 4 chondrocyte clusters by Seurat unsupervised clustering; ProC proliferative cluster, EcmC extracellular matrix (ECM) cluster, MetC metabolic cluster, DegC degradative cluster. **c** t-SNE projection of cells and representative markers in 4 clusters. **d** Percentages of 4 clusters in different passages. **e** Heatmap of cluster-specific genes. **f** Relative expression level and percentage of representative markers in 4 clusters. **g** Enriched Gene Ontology (GO) terms in 4 clusters.

Col2-pd2EGFP reporter chondrocytes were used to examine the expression of the hyaline cartilage lineage marker *Col2a1* during P0–8.^34^ *Col2a1* expression decreased as the passage number increased, which indicated a reduction in the functional phenotype (Fig. S1C, E). The transcriptomes of P0–8 chondrocytes were analyzed by performing scRNA-seq using a Fluidigm^TM^ C1 workstation (Fig. 1a). After the removal of low-quality data, we obtained the profiles of 634 cells, which covered 19 P0, 369 P2, 187 P4, and 59 P8 chondrocytes. A total of 6.9 ×10^7^ reads and an average of 4 680 genes per cell were detected (GSE193742). The expression of representative dedifferentiation markers for P0–8 was detected and matched with the classical dedifferentiation patterns,^8^ including the reduced expression of cartilage functional markers (*Col2a1*, *Acan* and *Col11a2*) and the enhanced expression of fibrosis/degradation-related genes (*Col1a1*, *Mmp13* and *Mmp3*) (Fig. S1F).

As a small number of cells was used to examine the transcriptomes of P0–8 chondrocytes, the results were compared with previously published scRNA-seq data for mouse primary chondrocytes and fibroblasts (GSE118236) (Fig. S1G).^35^ The principal component analysis (PCA) plot showed that all chondrocyte populations were distinctively distributed from mouse fibroblasts. Most P0 cells (18/19) merged with mouse primary chondrocytes from another data resource (GSE118236)^35^ (Fig. S1G), indicating that the evaluated P0 chondrocytes could represent typical primary chondrocyte subpopulations. We also confirmed the chondrogenic identity in the detected cells by demonstrating the high level of *Col2a1/Col1a1* (ratio of chondrocyte marker expression to fibroblast marker) compared to that in mouse fibroblasts (Fig. S1H). This result was supported by the finding that the detection of chondrocyte dedifferentiation was not due to the overgrowth of fibroblasts present in the cultures.^8^ Overall, we established an in vitro cell model with typical phenotypic changes in chondrocyte dedifferentiation.

### ScRNA-seq identifies distinct cell subpopulations in the early and late passages

Next, we analyzed chondrocyte subpopulations in P0–8 cells using Seurat unsupervised clustering from the R package.^36^ Four chondrocyte clusters were distinctly classified (Fig. 1b), with over 200 cluster-specific genes on average for each cluster (Fig. 1e, f, Table S1). We termed these clusters using Gene Ontology (GO) analysis (Fig. 1b). Cluster 1 was named the proliferative cluster (ProC, Fig. 1b) and was characterized by proliferative genes (*Mki67* and *Top2a*, Fig. 1c, e, f). The ProC was enriched for GO terms that included the *cell cycle* and *DNA replication* (Fig. 1g). Cluster 2 was defined as the ECM cluster (EcmC), and it was characterized by highly expressed cartilage ECM genes, including *Col9a1*, *Col11a2*, and *Col9a3* (Fig. 1c, e, f). The cluster was enriched for GO terms related to matrix production, including *tRNA aminoacylation* and *proteinaceous ECM* (Fig. 1g).

Cluster 3 was referred to as the metabolic cluster (MetC) and was characterized by metabolic-associated genes, retinol-binding protein 4 (*Rbp4*), isocitrate dehydrogenase *1* (*Idh1*), and secretory leukocyte peptidase inhibitor (*Slpi*) (Fig. 1c, e, f) and GO terms, including *extracellular region*, *growth factor activity*, and *mitochondria*. Cluster 4 was termed the degradative cluster (DegC) and contained metalloendopeptidase genes (*Mmp3* and *Mmp13*, Fig. 1f). The GO terms *extracellular matrix*, *actin cytoskeleton*, and *positive regulation of cell migration* were enriched in the DegC (Fig. 1g).

To infer the dedifferentiation stages of each cluster, we plotted the cluster proportion of P0–8 (Fig. 1d). The ProC was mainly detected in P0 and P2 (42.1% of P0 and 26.9% of P2) (Fig. 1d). The EcmC had a similar distribution to the ProC (57.9% of P0 and 37.8% of P2) (Fig. 1d), which is likely to represent primary chondrocytes with abundant ECM features. The MetC was not detected in P0 and occupied less than 10% of P4 and P8; however, it accounted for 37.8% of P2 (Fig. 1d) and thus was assumed to be an early dedifferentiated cluster. The DegC was present in the majority of P4 and P8 (77.1% of P4 and 74.6% of P8) (Fig. 1d), which suggested that the DegC was a late-dedifferentiated cluster.

### Pseudotime analysis reconstructs a loss-of-function trajectory

**Fig. 2:**
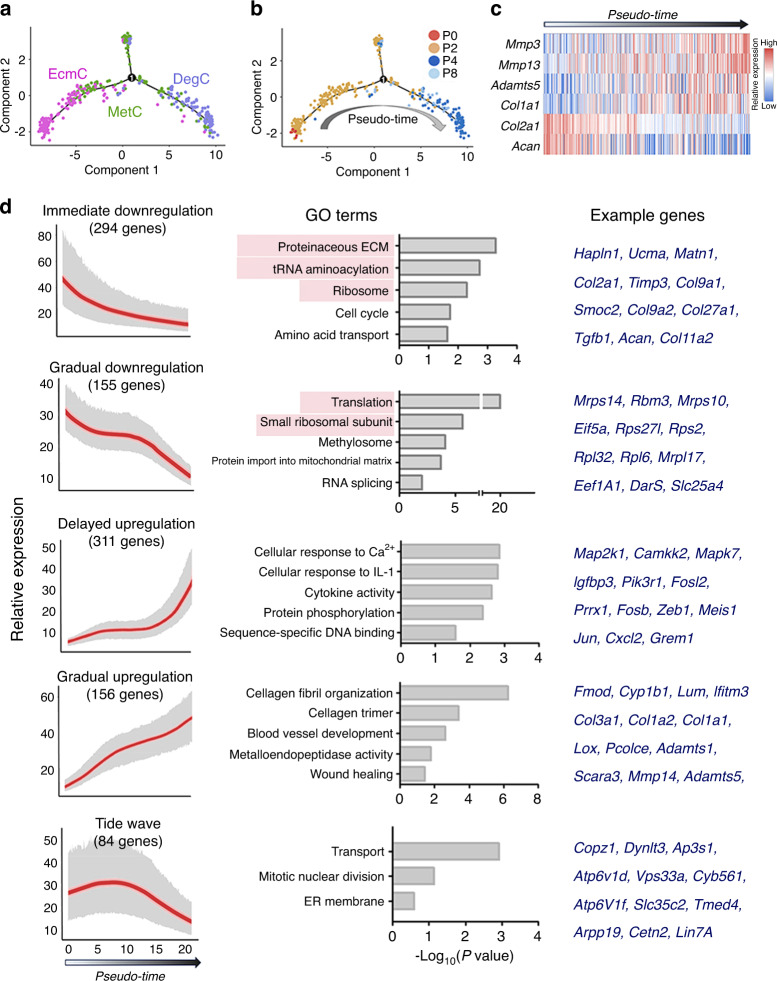
Pseudotime analysis reconstructs a loss-of-function trajectory. **a** Pseudotime trajectory profiles of cells in the EcmC, MetC, and DegC revealed in a two-dimensional independent componentspace, reconstructed by Monocle. **b** Pseudotime trajectory profiles of cells in the EcmC, MetC, and DegC marked by passages. **c** Heatmap of representative dedifferentiation markers expressed in pseudotemporal order. **d** Expression pattern (left), enriched GOterms (middle) and examples (right) of dynamically regulated genes based on pseudotemporal order: immediately downregulated, gradually downregulated, delayed upregulated, gradually upregulated and tide wave style genes.

We reconstructed a pseudotemporal trajectory based on cell-to-cell similarity using the R package Monocle^37^ (Fig. 2a, b). Consistent with our previous hypothesis, EcmC cells were aligned to the beginning of the trajectory, while DegC cells were mostly aligned to the late stage. The unique MetC, which represented the early dedifferentiated stage, was predominantly postulated to be present in the midterm of the trajectory (Fig. 2a, b). In addition, we showed the patterns of the dedifferentiation and cartilage functional biomarkers (*Mmp3*, *Mmp13*, *Adamts5*, *Col1a1*, *Col2a1* and *Acan*) on the trajectory axis as controls (Fig. 2c).

Next, we systemically characterized the time-lapsed transcriptome patterns by listing the top 1 000 genes that were dynamically regulated (*q* < 0.01). The genes were classified into five groups based on their expression patterns (Fig. 2d, Table S2): immediate downregulation, gradual downregulation, delayed upregulation, gradual upregulation, and tide wave pattern. Briefly, genes that were expressed early and showed immediately downregulated expression were assigned to GO terms that included *proteinaceous ECM*, *tRNA aminoacylation*, and *ribosome* (Fig. 2d). Genes that showed gradually downregulated expression were aligned with the GO terms *translation*, *small ribosome subunit*, *methylosome*, and *protein import into the mitochondrial matrix* (Fig. 2d). In contrast, genes with delayed upregulated expression were involved in the inflammatory responses *cellular responses to calcium (Ca*^*2+*^*)*, *cellular responses to interleukin (IL)-1*, and *cytokine activity* (Fig. 2d). Genes involved in *collagen fibril organization*, *collagen trimer*, *blood vessel development*, and *metalloendopeptidase activity* showed gradually upregulated expression during chondrocyte dedifferentiation (Fig. 2d). Transcription factor (TF)-coding genes with upregulated or downregulated expression were also detected (Table S3), and those regulating *transcription*, *Ca*^*2*+^ *responses*, *skeleton morphologies*, and *cAMP responses*, including *Jun* (C-JUN, AP-1 transcription factor subunit), *Fos*, *Runx1*, and *Creb3l1* (c-AMP responsive element binding protein 3 like 1), were highly expressed at the late stages (Fig. S2A, B). The TF genes with downregulated expression were involved in DNA binding and DNA stability^38^ and were associated with the minichromosome maintenance complex (Fig. S2A, C).

In all, genes responsible for ECM production and protein translation showed widely downregulated expression during chondrocyte dedifferentiation, while the genes for stress, inflammatory responses, and collagen reorganization showed significantly upregulated expression.

### Early dedifferentiated chondrocytes highly express a wide spectrum of metabolic genes

**Fig. 3:**
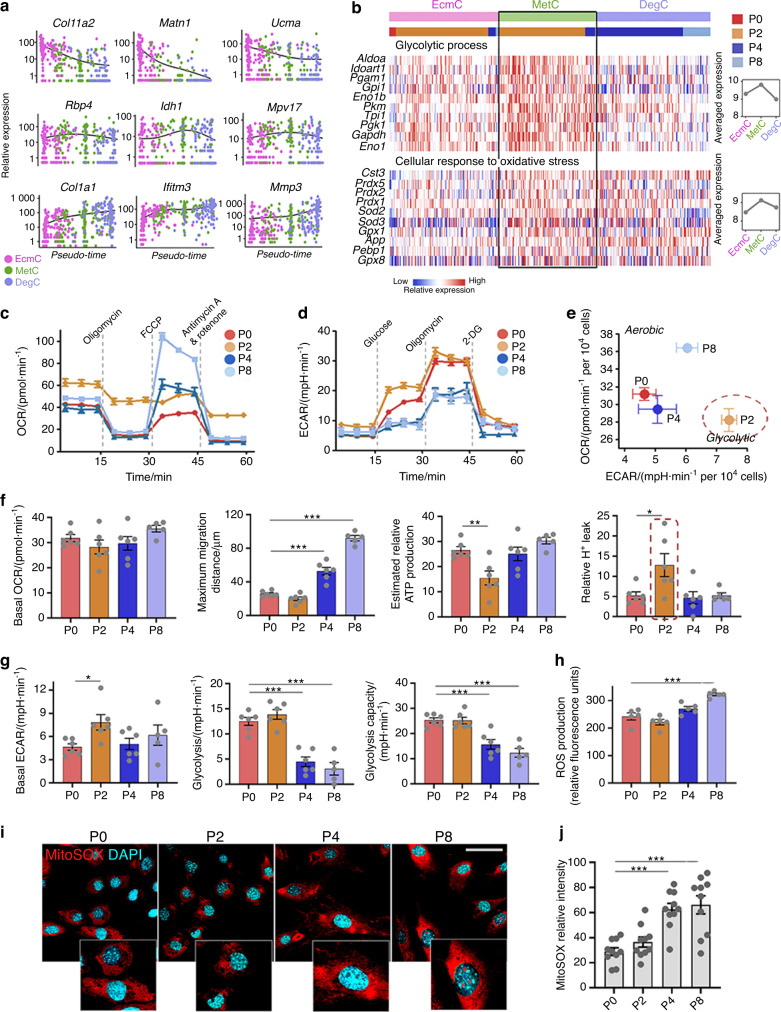
Early dedifferentiated chondrocytes highly express metabolic genes and obtain a glycolytic phenotype. **a** Typical cluster marker expression based on the pseudotemporal order. **b** Representative genes of *glycolytic processes* and *cellular responses to oxidative stress* in the EcmC, MetC, and DegC. **c** Oxygen consumption rate (OCR) assays of P0-8 chondrocytes. **d** Extracellular acidification rate (ECAR) analysis of P0-8 chondrocytes. **e** Two-dimensional bioenergetic profiles of P0-8 chondrocytes. **f**, **g** Other test parameters of OCR and ECAR assays of P0-8 chondrocytes. **h** Relative total intracellular ROS production in P0-8 chondrocytes. **i**, **j** Relative mitochondrial ROS production in P0-8 chondrocytes and the quantitative data, detected by MitoSOX Red staining; scale bars: 50 μm. All data are the mean ± SEM. *P < 0.05, **P < 0.01, ***P < 0.001.

We further characterized early dedifferentiated chondrocytes. The cluster distribution indicated that P2 cells partially retained the features of P0 chondrocytes (ProC and EcmC) (Fig. 1d) but also contained the unique subpopulation MetC, which mostly represented early dedifferentiated chondrocytes (Fig. 2a). The MetC was enriched for metabolism-associated genes, including *Rbp4* (encoding adipokine retinol-binding protein 4), *Idh1* (encoding isocitrate dehydrogenase 1) and *Mpv17* (encoding mitochondrial inner membrane protein 17) (Fig. 3a).

The GO terms enriched in the MetC relative to the EcmC included *extracellular region*, *sterol biosynthetic process*, *chemotaxis*, *glutathione transferase activity*, and *mitochondrial outer membrane* (Fig. S3B). KEGG pathway analysis suggested that genes assigned to the *cytochrome P450 metabolism* and *glutathione metabolism* pathways had significantly upregulated expression in the MetC (Fig. S3C), with the representative genes being *Mgst1*, *Mgst3* (genes encoding microsomal glutathione S-transferase 1 and 3), *Gstt1*, *Gstt2* (genes encoding glutathione S-transferase theta 1 and 2), *Cyp51* (cytochrome P450, family 51), and *Sod3* (superoxide dismutase 3) (Fig. S3D). Furthermore, we revealed that genes involved in the *glycolytic process* (*Cst3*, *Prdx5*, *Sod3*, *Gpx1* and *Pebp1*, etc.) and *cellular response to oxidative stress* (*Aldoa*, *Gpi1*, *Pgk1*, *Gapdh* and *Eno1*, etc.) were activated in the MeC compared to the EcmC and DegC (Fig. 3b). Thus, the major transcriptional features of the MetC have been demonstrated to be involved in metabolic processes.

In addition, genes associated with chemotaxis and chondrocyte hypertrophy were highly expressed in the MetC (Fig. S3E, G). Consistently, approximately 39% of P2 chondrocytes were shown to have a migratory phenotype with a fibroblast-like shape in comparison with the typical round shape predominantly present in primary chondrocytes (Figs. S1A and S3H). Genes associated with proliferation did not show significantly upregulated expression in the MetC (Fig. S3F). Thus, the results suggested that the genes typically expressed in the early dedifferentiated cluster are associated with metabolic activation, chemotaxis, and chondrocyte hypertrophy but not proliferation.

### Early dedifferentiated chondrocytes show a glycolytic phenotype during metabolic reprogramming

The unique subpopulation MetC, characterized by metabolic activation, occupied distinct proportions in different passages (Fig. 1d) and likely represented early differentiated chondrocytes. Thus, we further examined whether metabolic activation could be validated with cellular metabolic behavior. We performed live-cell metabolic assays to measure the oxygen consumption rate (OCR) and extracellular acidification rate (ECAR) in P0–8 chondrocytes (Fig. 3c–g). Briefly, the results indicated an anaerobic-to-aerobic metabolism switch during chondrocyte dedifferentiation (Fig. 3c–g). P0 and P2 cells had a significantly higher glycolytic rate and capacity than P4 and P8 cells (Fig. 3d, g, *P* < 0.001), while P4 and P8 cells showed increased maximal OCR respiration after FCCP injection (*P* < 0.001), suggesting an enhanced oxidative phosphorylation capacity to meet the increased energy demand (Fig. 3c, f). Increased levels of intracellular total reactive oxygen species (ROS) were also detected in later passages, especially in P8 chondrocytes (Fig. 3h and Fig. S3J, *P* < 0.001). The analysis specifically measuring mitochondrial ROS showed that late-stage chondrocytes had an increased level of mitochondrial ROS, in a similar trend to that of total ROS (Fig. 3i, j).

Notably, P2 cells showed a glycolytic phenotype when compared to cells at other passages, according to the metabolic phenotype map (Fig. 3e), and had a higher level of basal ECAR and glycolysis rate than P0 cells (Fig. 3g). The OCR results demonstrated that P2 chondrocytes produced less mitochondrial ATP than the cells from other passages, with more proton (H^+^) leakage uncoupled to ATP production (Fig. 3f). Increased H^+^ leakage has been documented as a protective mechanism against oxidative stress.^39,40^ Consistent with this finding, ROS production did not significantly increase in P2 chondrocytes compared to P0 chondrocytes (Fig. 3h, Fig. S3J, I, J).

Overall, live-cell metabolic assays demonstrated that dedifferentiated chondrocytes underwent a metabolic transition with a higher aerobic respiration capacity. However, P2 cells showed a unique glycolytic metabolic phenotype with increased mitochondrial proton uncoupling and reduced ROS than the cells at other passages. This finding was consistent with the result that P2 contained the highest population of the MetC, a cluster with activated genes for *the glycolytic process* and *cellular response to oxidative stress*.

### Late dedifferentiated chondrocytes are characterized by ultrastructural changes of metabolic stress

Next, we focused on late-dedifferentiated chondrocytes. The DegC represented the majority of P4 and P8 chondrocytes (Fig. S4A, B) and contained genes involved in cytoskeleton reorganization, matrix degradation, inflammatory response, and chondrocyte hypertrophy (Fig. 1g, Fig. S3G, I). In particular, the DegC was also characterized by features of mitochondrial stress, as well as the mitochondrial unfolded protein response,^41,42^ a self-protective mechanism that promotes cell survival against external stimuli and delays mitochondrial dysfunction.^41,43–45^ Relevant characteristics for the mitochondrial unfolded protein response, including attenuated translation, impaired mitochondrial homeostasis, activated mitochondrion-to-nucleus retrograde pathways, and epigenetic alterations, have been reported.^43,44,46^ Consistently, in our results, the number of genes expressed per cell was reduced in the DegC (Fig. S4C), consistent with a previous study,^18^ and the lowest expression of translation-relevant genes was detected (Fig. S4D). Genes related to mitochondrial function (translocase and porins), including *Timm17a*, *Timm23*, *Tomm22*, *Vdac2* and *Vdac3*, showed slightly upregulated expression in the MetC but downregulated expression in the DegC (Fig. S4D). Genes of the MAPK pathway and AP-1 family that are involved in mitochondrial retrograde signaling^47,48^ and are known nuclear responsive factors were highly expressed in the DegC (Fig. S4D).

**Fig. 4:**
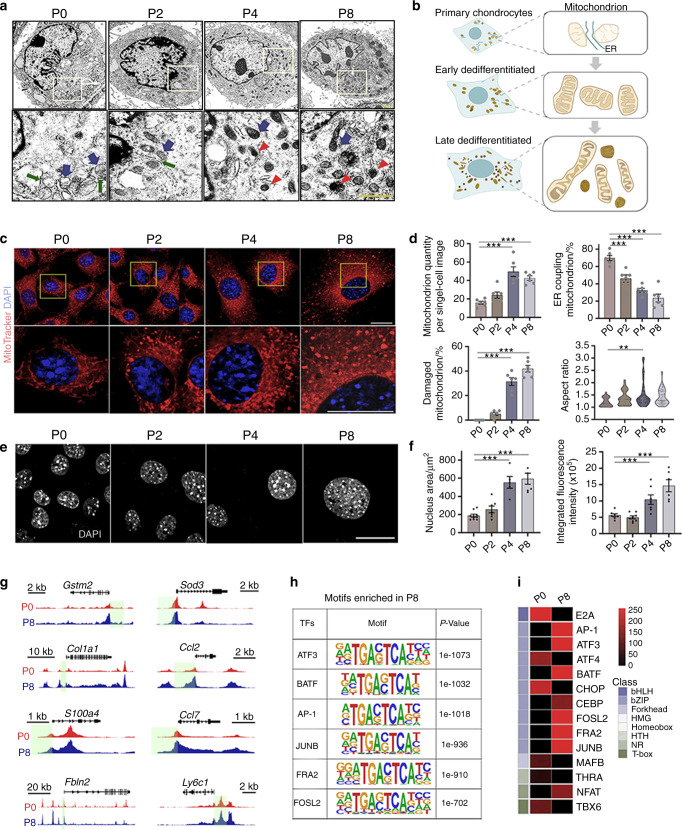
Late dedifferentiated chondrocytes exhibit ultrastructural changes in metabolic stress. **a** Representative images of mitochondria in P0-8 chondrocytes by transmission electron microscopy; scale bars: 1 μm; blue arrows: mitochondria, red arrows: damaged mitochondria, green arrows: endoplasmic reticulum. **b** Schematic diagram of mitochondrial morphological features in primary (P0), early dedifferentiated (P2) and late dedifferentiated chondrocytes (P4 and P8). **c** Representative images of MitoTracker-stained mitochondria in P0-8 chondrocytes; scale bars: 20 μm. **d** Quantitative analysis of mitochondrial TEM images of P0-8 chondrocytes. **e**, **f** Representative images and quantitative analysis of DAPI-stained nuclei in P0-8 chondrocytes by confocal microscopy; scale bars: 20 μm. **g** Representative genes with P8-specific open regions in promoters (promotor regions are marked in green). **h** Representative enriched TF binding motifs in P8. **i** Heatmap of enriched motifs in P0 and P8. All data are the mean ± SEM. *P < 0.05, **P < 0.01, ***P < 0.001.

To confirm impaired mitochondrial homeostasis in late dedifferentiated chondrocytes, we examined the ultrastructure of P0-8 chondrocytes by transmission electron microscopy (TEM) (Fig. 4a). Quantitative analysis demonstrated an increased number of mitochondria in P4 and P8 cells (Fig. 4a, d), matching their aerobic phenotype. Specifically, P0 displayed more homogeneous and round mitochondria, with lower aspect ratios (Fig. 4a, b, d), while P2 mitochondria were relatively elongated (Fig. 4a, b, d) compared to those of other groups. In contrast, P4 and P8 contained perinuclear, heterogeneous mitochondria with disorganized configuration and an electron-dense damaged structure^49^ (red arrows) (Fig. 4a, b, d). Similarly, MitoTracker images showed swelling and fragmentation of mitochondria in late dedifferentiated cells (Fig. 4c).

Overall, the results showed that late dedifferentiated chondrocytes contained an increasing quantity of mitochondria to meet metabolic demand. However, structural damage to the mitochondria was observed. As mitochondrial morphology is controlled by metabolism,^50^ this finding may indicate the dynamic fusion and fission of mitochondria.

### Late dedifferentiated chondrocytes exhibit stress-associated chromatin remodeling

Together with the organelle structure, nuclear morphology changed dramatically with increased passage number. High-resolution confocal images showed that nuclei in P4 and P8 chondrocytes were significantly enlarged with enhanced integrated fluorescence intensity (Fig. 4e, f), suggesting a structural alteration in nuclear architecture and chromatin condensation.^51^

Next, an assay for transposase-accessible chromatin with high throughput sequencing (ATAC-seq) was performed based on a previous method^52^ (P0 *vs*. P8, 2 replicates for each, Fig. S4E) to confirm the chromatin landscape modifications in late-dedifferentiated chondrocytes. A decrease in global chromatin accessibility was detected in P8 chondrocytes (Fig. S4F, GSE193743), and stage-specific peaks at 16754 and 5971 were identified at P0 and P8, respectively (Fig. S4G). P8 cells were shown to have fewer open regions near gene promoters (<1 kb) and fewer TF binding loci near transcriptional start sites (TSS, <1 kb) (Fig. S4I–L). In contrast, the proportion of distal intergenic regions in P8 cells decreased from 43.3% to 37.5% in P0 cells (Fig. S4K).

Interestingly, similar epigenetic alterations have been reported in stress responses of other lineages.^53,54^ The transcription of most cellular genes was disrupted, but specific downstream effectors were activated. Specifically, we identified representative P8-specific regions with enhanced accessibility near promoters and found that the genes associated with these regions were relevant to fibrosis (*Col1a1*, *S100a4*, and *Fbln2*), immune responses (*Ccl2*, *Ccl2* and *Ly6c1*), and metabolic stress (*Gstm2* and *Sod3*) (Fig. 4g). We also compared the TF binding motifs in P0 and P8 cells (Fig. 4h, i). The data revealed that binding sites for TFs, which play essential roles in mitochondrion-to-nucleus signaling, oxidative stress, and inflammatory responses, including the ATF3, BATF, and AP-1 family, were strongly enriched in P8 cells (Fig. 4h, i).

Thus, late dedifferentiated chondrocytes exhibited epigenetic remodeling with a global decrease in chromatin accessibility and increased open regions in genes responsive to metabolic stress.

### Manipulating mitochondrial F1Fo-ATPase efficiently ameliorates early dedifferentiation

**Fig. 5:**
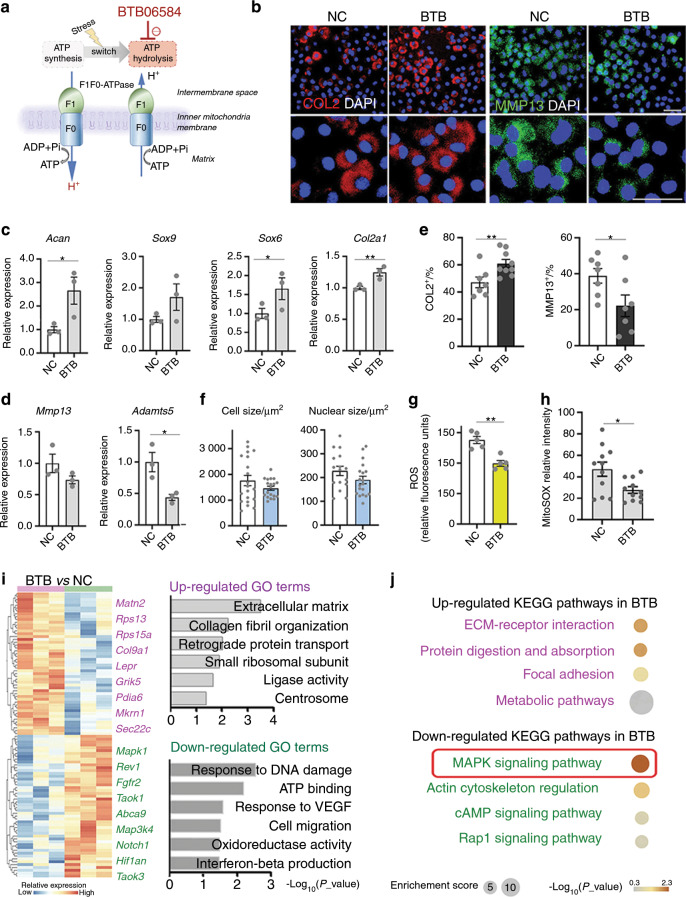
Manipulating mitochondrial F1F0ATPase efficiently ameliorates early dedifferentiation. **a** Schematic diagram of BTB06584 (BTB)’s effect on F1Fo-ATPase. **b** Representative immunostaining images of COL2 and MMP13 in the BTB-untreated (negative control, NC) and treated P2 chondrocytes; scale bars: 50 μm. **c**, **d** qPCR detection of representative dedifferentiation genes in the NC and BTB-treated P2 chondrocytes. **e** Quantitative data of COL2 and MMP13 immunostaining of the NC and BTB-treated P2 chondrocytes. **f** Quantitative analysis of the cellular and nuclear sizes of the NC and BTB-treated P2 chondrocytes. **g** Quantitative analysis of relative ROS production in the NC and BTB-treated P2 chondrocytes. **h** Quantitative analysis of relative mitochondrial ROS production in the NC and BTB-treated P2 chondrocytes. **i** Heatmap of differentially expressed genes and enriched GO terms in the BTB-treated *vs*. NC chondrocytes, detected by bulk RNA-seq. **j** Up- and downregulated KEGG pathways in the BTB-treated P2 *vs*. NC chondrocytes. The final concentration of BTB was 2.5 μmol·L^−1^. “NC” (negative control) in this figure represents the treatment with 0.025% dimethyl sulfoxide (DMSO), the solvent of BTB. All data are the mean ± SEM. *P < 0.05, **P < 0.01, ***P < 0.001.

Having identified the increasing metabolic stress and mitochondrial dysfunction during chondrocyte dedifferentiation, we hypothesized that manipulating stress signaling might influence the chondrocyte functional phenotype. F1Fo-ATPase is the prime ATP producer on the inner mitochondrial membrane and essentially regulates mitochondrial function.^55^ This molecule synthesizes ATP by pumping H^+^ across the inner mitochondrial membrane to the mitochondrial matrix. In some pathological events, when mitochondrial homeostasis is compromised, F1Fo-ATPase can degrade ATP and reverse the direction to generate proton backflow (from the mitochondrial matrix to the intermembrane space).^56–58^ The chemical compound BTB06584 (BTB) selectively inhibits F1Fo-ATPase ATP hydrolysis and reduces F1Fo-ATPase-driven H^+^ backflow without compromising ATP synthesis^58^ (Fig. 5a).

BTB treatment of P2 and P4 chondrocytes resulted in different phenotypic changes. Immunostained images demonstrated that BTB significantly promoted COL2 expression and inhibited MMP13 expression in P2 chondrocytes (Fig. 5b, e). We then conducted real-time qPCR, and the results showed higher expression levels of representative functional genes (*Acan*, *Sox6*, *Sox9* and *Col2a1*) and suppression of matrix degradation genes (*Adamts5* and *Mmp13*) in the BTB-treated group (Fig. 5c, d). The BTB-treated P2 chondrocytes did not exhibit distinct cellular or nuclear sizes (Fig. 5f). We also compared the phenotypes of the BTB-treated P2 cells with those of the BTB-untreated P1 and P2 chondrocytes. The immunostaining results of COL2, ACAN and MMP13 demonstrated that BTB treatment could not fully revert P2 to P1 but rescued most of the phenotype loss (Fig. S5A, B).

In live-cell metabolic assays, P2 cells showed a high level of H^+^ leakage into the mitochondrial matrix, which was assumed to be due to ROS production,^39^ one of the triggers of mitochondrial-mediated stress.^59–62^ As BTB inhibited ATP hydrolysis and thereby reduced H^+^ backflow, we hypothesized that BTB's effect might be associated with decreased ROS production in P2 chondrocytes. We measured both total intracellular and mitochondrial ROS (Fig. 5g, h, Fig. S5D, E). Consistently, the results showed that the BTB-treated P2 chondrocytes significantly produced less ROS. No morphological difference was observed in the mitochondria of the BTB-treated and control P2 cells (Fig. S5C).

In P4 cells representing late dedifferentiated chondrocytes, BTB significantly inhibited MMP13 expression but had no effect on COL2 expression (Fig. S6A, B). BTB significantly inhibited only the matrix degradation genes *ADAMTS5* and *MMP13* (Fig. S6C, D). The BTB-treated P4 chondrocytes had a relatively smaller cellular size (Fig. S6G) but a similar nuclear size to the control group (Fig. S6G). Moreover, in P4 chondrocytes, ROS production was not suppressed by BTB (Fig. S6E, F), and the fragmentation and swelling of mitochondria was not significantly altered (Fig. S6H). TEM revealed that damaged and heterogeneous mitochondria were not obviously reduced in the BTB-treated P4 cells (Fig. S6I).

Thus, we revealed that the chemical inhibitor BTB could effectively ameliorate functional phenotype loss in early dedifferentiated chondrocytes (P2) but not in late dedifferentiated chondrocytes (P4). ROS production could be significantly suppressed in P2 chondrocytes. The mitochondrial structural damage in P4 cells was not obviously ameliorated.

### BTB ameliorates early phenotype loss via the MAPK pathway

As BTB treatment had a suggested therapeutic effect during chondrocyte dedifferentiation, especially at early passages, we performed bulk RNA-seq to further investigate the mechanisms by which BTB ameliorates P2 chondrocyte phenotype loss (Table S4). The highly expressed genes in the BTB-treated chondrocytes were aligned with the following GO terms: *extracellular matrix*, *collagen fibril organization*, *retrograde protein transport*, and *small ribosomal subunit* (Fig. 5i).

The representative genes with upregulated expression were shown to be responsible for ECM formation and included *Matn2*, *Col9a1*, *Rps13* and *Rps15a*. The suppressed genes were related to the GO terms *DNA damage response*, *ATP binding*, *vascular endothelial growth factor (VEGF) response*, and *cell migration* (Fig. 5i), which had been identified as dedifferentiated signatures (Figs. 1g and 2d).

Additionally, KEGG analysis demonstrated that the MAPK pathway was mostly decreased in the BTB-treated P2 chondrocytes (Fig. 5j). Typically, the expression levels of *Mapk1*, *Map2k4*, and *Map3k4* were significantly inhibited by BTB treatment, and the corresponding nuclear responsive genes that mediate the downstream reaction (*Jun*, *Creb3l2*, *Nfkb2* and *Atf2*) were significantly inhibited by BTB treatment (Fig. S5F). We further examined the activity of representative molecules in this pathway by Western blotting (Fig. S5G). The results demonstrated that the protein expression level and phosphorylation status of p38 MAPK and c-Jun were suppressed by BTB, and those of p42/44 MAPK were sustained (Fig. S5G). Interestingly, our data showed that the pathway was enhanced in late dedifferentiation (Fig. S4D), similar to those in other studies of chondrocyte dedifferentiation.^18^ The MAPK pathway has also been identified as a mitochondrion-to-nuclear retrograde signaling pathway in metabolic stress,^48^ which could be activated by ROS.^63^

Conversely, the expression of the mitochondrial functional markers *VDAC2* and lactate dehydrogenase A (*LDHA*) was elevated (Fig. S5F). Representative genes with upregulated expression in the MetC that were assigned to the *glycolytic process*, *gluconeogenesis*, and *cellular response to oxidative stress* processes (Fig. 3b) were not significantly altered by BTB (Fig. S5F).

Overall, we demonstrated a potential mechanism of ameliorating early dedifferentiation with BTB treatment, involving a reduced production of ROS and a regulation of the mitochondrial retrograde signaling MAPK pathway.

### The two-stage transition is validated in the human chondrocyte dedifferentiation model

**Fig. 6:**
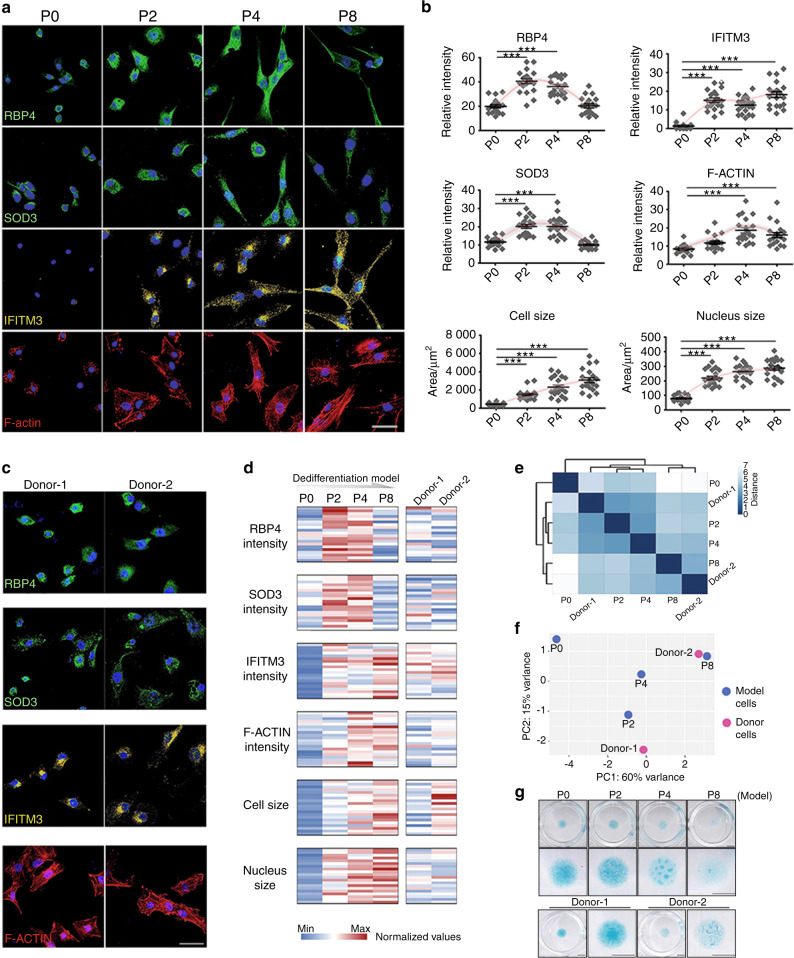
Early and late dedifferentiation biomarkers predict human chondrocyte plasticity. **a** Confirmation of the immunostained scRNA-seq-defined markers RBP4, SOD3, IFITM3, and F-actin in human P0-8 chondrocytes, as a human chondrocyte dedifferentiation model; scale bars: 50 μm. **b** Quantitative data of immunostained scRNA-seq-defined markers, cell/nucleus size and cell relative viability in human P0-8 chondrocytes. **c** Immunostained RBP4, SOD3, IFITM3, and F-actin in human articular chondrocytes from 2 other donors (Donor 1 and 2) for phenotype evaluation; scale bars: 50 μm. **d** Heatmap of captured parameters in the human chondrocyte dedifferentiation model and articular chondrocyte samples from 2 donors, including immunostained RBP4, SOD3, IFITM3 and F-actin intensity, as well as cell size and nucleus size. Each cell represents the detection of one biomarker in one cell. Each row represents a round of detection. Twenty cells were detected for one biomarker in each batch. **e**, **f** Sample-to-sample distance heatmap and principal component analysis (PCA) plot showing the similarity of Donor-1 and Donor-2 chondrocytes with the human chondrocyte dedifferentiation model (P0-8). **g** Alcian blue–stained micromasses formed by the human chondrocyte dedifferentiation model (P0-8) and Donor-1 and 2 chondrocytes; scale bars: 3 mm. All data are the mean ± SEM. *P < 0.05, **P < 0.01, ***P < 0.001.

The different effects of BTB on P2 and P4 chondrocytes indicated that early and late dedifferentiated chondrocytes possess unequal potential to recover from functional loss. Using continuously passaged mouse and human chondrocytes (polydactyly chondrocytes, Table S5, age 9–24 m, *n* = 6),^64^ we confirmed the phenotypic transition by examining the protein levels of RNA-defined markers (Fig. S7A and Fig. 6a), including RBP4, SOD3 (both MetC markers in early dedifferentiation, Fig. 1f), IFITM3 (interferon-induced transmembrane protein 3, the corresponding gene gradually increased in the pseudotime trajectory, Fig. 2d) and filamentous actin (F-actin, a defined DegC feature in late dedifferentiation, Fig. 1g). Consistently, RBP4 and SOD3 were highly expressed in the middle of the serial culture (P2 in the murine model and P2/4 in the human model), while the expression of IFITM3 and F-actin was gradually upregulated (Figs. S7A, S7B, and Fig. 6a, b).

Similarly, in mice and humans, late dedifferentiated chondrocytes exhibited a fibroblast-like morphology with strong F-actin staining (Fig. S7A and Fig. 6a). The cell/nucleus size increased during the dedifferentiation of chondrocytes (Figs. S1B, Figs. 4f and 6b). Although the rate of dedifferentiation may vary among different species, individuals, and culture conditions, a two-stage transition in a human chondrocyte dedifferentiation model was also observed.

**Fig. 7:**
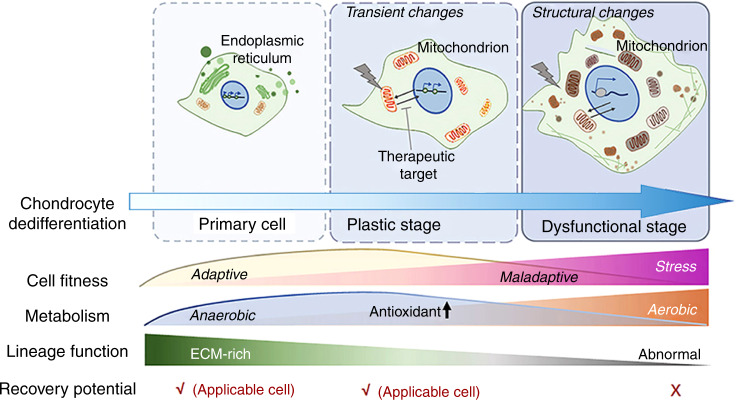
The biphasic model of chondrocyte dedifferentiation. The schematic diagram of biphasic chondrocyte dedifferentiation model. Early-stage chondrocytes transiently produce less ECM and exhibit an increased expression of anti-oxidative genes. Late-stage chondrocytes exhibit structural changes involving mitochondrial damage and stress -associated chromatin remodeling. Early stage (plastic stage) reserves the potential to be rescued from functional loss but late stage (dysfunctional stage) does not.

### Early and late dedifferentiation biomarkers predict human chondrocyte plasticity

In clinical trials, the cell passage number cannot fully reflect cell quality. Therefore, to evaluate the human chondrocyte phenotype, we established a platform to examine the degree of human chondrocyte dedifferentiation based on visual indicators previously defined in our scRNA-seq data and tested this system with articular chondrocytes extracted from different donors. The calculated parameters corresponded to the expression of RBP4, SOD3, IFTIM3, and F-actin, the cell/nuclei size, and cell viability. Relative intensity values were calculated for 20 randomly selected individual cells, and the cell/nuclei size was measured based on phalloidin and DAPI staining (Fig. S8A).

Based on the captured parameters, we compared the quality of chondrocytes from two different human donors with distinctive backgrounds (Table S5) using the DESeq2 sample distance measurement. In the sample-to-sample distance heatmap and principal component analysis (PCA) plotting, donor-1 chondrocytes were plotted close to P2 cells, while donor-2 cells were more similar to P8 cells (Fig. 6e, f). This finding indicated that donor-1 chondrocytes were at a relatively early stage, while donor-2 cells were predicted to have a phenotype similar to that of late dedifferentiated chondrocytes. To confirm the calculation of the sample-to-sample distance, we randomly reordered the captured values in several rounds of detection and found that similar outcomes were still presented in 3 different batches (Fig. S8D).

To further validate this prediction, we reseeded cells of donors 1 and 2 in a 3D micromass to examine their potential chondrogenic capacity (Fig. 6g). The micromass of donor-1 produced strongly stained cartilage-like ECM similar to P0 and P2 micromasses, but donor-2 chondrocytes failed to recover in 3D culture and produced lightly stained ECM and were more similar to the P4 and P8 micromasses (Fig. 6g and Fig. S8C). According to the donor information (Table S5), donor-1 chondrocytes were collected from a 17-week-old fetus, whereas donor-2 chondrocytes were isolated from a 55-year-old OA patient.

We further verified that this system could sensitively evaluate chondrocytes from different backgrounds by including additional donors (Table S5, age 50–75 y, *n* = 4). P2 polydactyly chondrocytes were also used as a healthy control (Model-P2). Based on the calculation of 6 defined indicators (Fig. S9A, C), the sample-to-sample distance heatmap showed that cells from donor 3 had a higher correlation with P2 model chondrocytes compared to those from donors 4, 5 and 6 (Fig. S9D).

After normalization, PCA revealed the overall distribution of all detected samples (Fig. S9E). P2 model cells from two batches were located close together (Fig. S9E). Cells from donors 1, 3 and 4 were shown to be similar to P2 model chondrocytes (Fig. S9E), indicating that they were at an earlier stage than those of donors 2, 5 and 6. Donor-2 cells were still connected to P8 model chondrocytes. Chondrocytes from donors 5 and 6 were relatively far from P2 cells but were also not close to P0, P4 or P8 cells (Fig. S9E). Consistent with the PCA plotting, the micromass test demonstrated that cells from donors 3 and 4 produced comparable ECM with model P2 chondrocytes, while cells from donors 5 and 6 formed inferior micromasses (Fig. S9B, F).

Briefly, chondrocytes that were found to be at an early stage in the image-based evaluation (donors 1, 3, and 4) formed strongly stained micromasses (Fig. 6g, Figs. S8C, S9B, F). In contrast, those of donors 2, 5 and 6 were located far from P2 chondrocytes (Fig. S9E), and they consistently obtained lower ranks in the micromass test (Fig. 6g, Figs. S8C and S9B, F). However, only donor-2 cells had a tight correlation with late dedifferentiation (Fig. 6f and Fig. S9E). Donor-5 and -6 cells were not close to any other groups (Fig. S9E). This finding might have resulted from the heterogeneity of human chondrocytes, according to the high variation in the quantitative analysis of late-stage markers (IFITM3, F-actin and cell size), especially in donor-6 cells (Fig. S9A). This result suggested that the current panel could predict good-quality chondrocytes but only provided a rough indication for the samples that were not at an early stage.

In summary, we validated the biphasic transition in human chondrocytes (Fig. 7) and established an image-based system to predict chondrocyte function using scRNA-seq-defined biomarkers. Thus, the time-lapse dedifferentiation atlas will not only uncover stepwise phenotype dynamics in murine and human chondrocyte dedifferentiation but will also provide a database for cell quality prediction.

## Discussion

Manipulation of the chondrocyte phenotype is a challenge, as the concept of chondrocyte dedifferentiation remains largely unknown. Here, we reconstructed a single-cell time-lapse atlas of chondrocyte dedifferentiation by identifying unique early and late dedifferentiated stages with distinct functional and metabolic phenotypes. We confirmed the stage biomarkers in mouse and human chondrocytes, providing a new perspective for cell-based cartilage regeneration.

This study can provide molecular details for the concept of chondrocyte dedifferentiation. Chondrocytes are the main cell type in cartilage and are responsible for ECM secretion to supply mechanical cushions and lubrication.^65^ In the pseudotime trajectory, ECM synthesis and translation-related genes were globally inhibited, which finely illustrated the transcriptional events causing chondrocyte function loss in the dedifferentiation process. Given that tissue function loss also occurs in other lineages,^11,12^ this study provides an in-depth understanding of pathological cell dedifferentiation. Our data revealed an end stage of chondrocyte dedifferentiation exhibiting subcellular structural changes in metabolic stress. The final state of the model resembles pathological hypertrophy, an adaptive response during tissue repair that involves compensatory growth without cell identity conversion.^66,67^ The similarities were indicated by the features presented, including^68–70^ (1) impaired tissue function, as shown by decreased ECM secretion in late dedifferentiated chondrocytes, (2) unbalanced protein synthesis and degradation, as shown by increased metalloendopeptidase activity at the late stage, (3) altered metabolism, as shown by enhanced ROS production in late dedifferentiated chondrocytes, and (4) an increased inflammatory response, as demonstrated by the GO terms that were related to the immune response at the late stage. Therefore, our findings help to clarify the cellular programs involved in chondrocyte dedifferentiation, which are similar to those in pathological hypertrophy. Additionally, cell dedifferentiation represents a reversion of mature cells to stem/progenitor cells in regenerative process in vivo.^67,71^ Although their correlations and differences may require further examination, this study still provides clues to distinguish their conceptions, as chondrocyte dedifferentiation in vitro was mainly documented as a loss-of-function process.^28^

Our model provides evidences to explain the biphasic switch whereby chondrocyte phenotypes can be rescued after a short-term monolayer culture but not long-term expansion.^72^ The data indicate that early chondrocyte dedifferentiation is a compensatory stage that compromises to produce less ECM but still reserves chondrogenic potential. This hypothesis is supported by the measurements of both tissue function and metabolic behavior. Early-stage chondrocytes formed ECM-rich micromass similar to that of primary chondrocytes. In metabolic assays, P2 cells presented a Warburg-like change with a higher glycolytic level. In other studies, the Warburg effect was shown to not only benefit stem cells^73,74^ but also be an early sign of metabolic stress in a nontumor disease.^75,76^ This phenomenon is because the Warburg effect maintains cellular energy levels with less ROS production and improves mitochondrion/tissue function.^43,77^ This result suggests that early-stage chondrocytes exhibit a compensatory response to energy metabolism.

Chondrocytes reside in a hypoxic environment in vivo, but evidence has shown that they are capable of using both anaerobic and aerobic metabolism to generate ATP,^78–80^ especially under some nonphysiological conditions. This paradox may be resolved considering that the TCA cycle is an essential source of intermediary metabolites and energy supply for supporting cell survival and tissue repair.^81^ In this pathological model, P2 cells exhibited an increased number of mitochondria but did not show damaged structures, as detected in P4 and P8 cells. The phenotypic loss of P2 chondrocytes was efficiently ameliorated by BTB, which inhibited ATP hydrolysis^58^ and suppressed the activation of the MAPK signaling pathway. These results indicate that early dedifferentiated chondrocytes still have the potential to restore cellular function without increased mitochondrial stress triggers. Nevertheless, BTB was not capable of reversing ultrastructural mitochondrial damage in P4 cells. The mechanisms of rescue failure in the late stage require further investigation and may be relevant to the severe functional loss in mitochondria, including the collapse of the mitochondrial membrane potential. Overall, our data illustrate a stepwise metabolic alteration in dedifferentiated chondrocytes, involving a switch from compensation to decompensation and changes from phenotype to ultrastructure. This study also highlights metabolic stress as a potential therapeutic target and the early compensatory stage as the optimal time for manipulation of the chondrocyte functional phenotype.

Chondrocyte dedifferentiation has severely compromised the outcomes of ACI,^7,20^ necessitating the development of a reliable chondrocyte prediction system. Our data provide informative resources for the identification of chondrocyte dedifferentiated phenotypes. We established an image-based evaluation system to illustrate the applicability of scRNA-defined biomarkers. Human chondrocytes from different donors could be conveniently distinguished by visual parameters, with a small number of cells required for expansion for each test. We collected transcriptomic features and verified them at the protein level in both murine and human models, indicating that dynamic changes in defined biomarkers exist in both mouse and human cells. However, human chondrocyte phenotypes vary with different donor individuals and tissue sites, hindering the analysis of common cellular regularities.^41^ Our evaluation could distinguish early dedifferentiated chondrocytes but did not sensitively identify the stage of chondrocytes with poor function. These heterogeneous chondrocytes did not uniformly highly express late-stage markers. There is scope for further optimization of the evaluation system and expansion of the indicator panel. Overall, our data showed the existence of a two-stage transition in human chondrocyte dedifferentiation and provided informative data resources for the development of a clinical cell evaluation system.

In conclusion, we constructed a high-resolution atlas of chondrocyte dedifferentiation and uncovered a stepwise metabolic adaptive program. The results of this study provide insights into chondrocyte function loss in vitro and lay a foundation for future regulation of clinical chondrocyte quality.

## Materials and methods

### Chondrocyte isolation and culture

Mouse chondrocytes were isolated from the knee cartilage of C57Bl/6 mice (postnatal day 0–4) following a previously published method.^32,33^ The digested cells were resuspended and cultured with DMEM/F12 medium (Gibco) with 10% fetal bovine serum at 37 °C with 5% CO_2_. P0 chondrocytes represent the primary chondrocytes that were cultured in vitro for less than 24 hours (h). For the dedifferentiation model, chondrocytes were passaged after doubling the cell quantity (seeding density: 2.4 × 10^4^ cells per cm^2^; collecting density: 4.8 × 10^4^ cells per cm^2^). Col2-pd2EGFP reporter chondrocytes^34^ were isolated from a strain of transgenic mice gifted by William A. Horton from Oregon Health and Science University. All animal experiments were approved by the Zhejiang University Ethics Committee (ZJU20160445).

Human chondrocytes used for the dedifferentiation model were isolated from polydactyly-derived cartilage (Table S5, *n* = 6, age 9–24 months old, 4 females, 2 males). The tissue was obtained from the uncalcified cartilage in joints removed during corrective surgery^64^ at the Children’s Hospital of Zhejiang University School of Medicine. Briefly, fresh cartilage was washed twice with PBS, cut into 1 mm3 slices and digested with 0.2% collagenase type II (Gibco) in serum-containing DMEM/F12 (Gibco) at 37 °C for 16 h. The digestion solution was filtered through a 40 µm filter to remove the minced tissue. Then, the cells were collected by centrifugation at 1 500 r·min^−1^ for 5 min and passaged for further use. This experiment was approved by the Ethics Committee of the Children’s Hospital of Zhejiang University School of Medicine (2020-IRB-007), and all individuals provided full written informed consent before the operative procedures.

The fetal human chondrocytes were isolated from the uncalcified cartilage in knee joints of a 17-week-old female fetus (Table S5). The digestion process was the same as that of polydactyly-derived chondrocytes. Chondrocytes at passage 2 were used in the study. This experiment was approved by the Research Ethics Committee of the First Affiliated Hospital, Zhejiang University (2018-115).

Adult human chondrocytes were obtained from undamaged articular cartilage obtained during surgical knee operations (Table S5, *n* = 5, age 55–75 years old, 2 females, 4 males). The fresh cartilage was washed with PBS, cut into 1 mm3 slices and digested with 0.2% collagenase type II (Gibco) and 0.15% collagenase type I (Gibco) in serum-containing DMEM/F12 (Gibco) at 37 °C for 16 h. The digestion solution was filtered through a 40 µm filter to remove the minced tissue. Then, the cells were collected by centrifugation at 1 500 r·min^−1^ for 5 min and passaged for further use. Chondrocytes at passage 2 were used in the study. This experiment was approved by the Ethics Committee of the Fourth Affiliated Hospital, Zhejiang University (K2021088).

### Single-cell transcriptomics

Single cells were captured using FluidigmTM C1 high-throughput IFC. CFDA-SE (Beyotime) dye was used to distinguish cells from different groups. Cells in every microfluidic chamber were assessed by microscopy for further data exclusion. Then, cell lysis, reverse transcription and cDNA preamplification were performed based on Fluidigm’s standard protocol. Libraries were pooled and sequenced 150 bp paired-end on one lane of Illumina HiSeq xten. The R package Seurat was used for dimension reduction, clustering and differential gene expression analysis.^36^ Briefly, we filtered out cells expressing <500 genes, resulting in 608 cells on average expressing 4 165 genes per cell. Dimensional reduction was performed with the highly variable genes, and significant principal components (*P* < 10^−7^) were used for unsupervised clustering. The R package Monocle^37^ was used for cell clustering and pseudotime analysis. Briefly, single-cell mRNA counts were loaded into Monocle as described by the package releasers (http://www.bioconductor.org/packages/release/bioc/html/monocle.html). Genes expressed by less than 10 cells were excluded, while qualified cells were chosen with total mRNA falling in the mean ±2 sd. The top 1 000 significant genes were chosen as ordering genes to define single-cell trajectories.

### Live-cell metabolic assay

The OCR and ECAR values were measured with a Seahorse XF96 extracellular flux analyzer (Seahorse Bioscience). Briefly, primary and passaged chondrocytes were seeded in an XF96 microplate at a density of 10^4^ cells per well and preincubated overnight. Before the assays, cells were equilibrated for 1 h in unbuffered XF assay medium (for OCR: base medium with 10 mmol·L^−1^ glucose, 2 mmol·L^−1^ glutamine and 1 mmol·L^−1^ pyruvate, pH 7.4; for ECAR: base medium with 1 mmol·L^−1^ glutamine). The XF Cell Mito Stress Test kit and XF Glycolysis Stress Test kit were used following standard protocols (https://www.agilent.com). During the assay, compounds were injected at the following final concentrations: 1 μmol·L^−1^ oligomycin, 1 μmol·L^−1^ FCCP and 1 μmol·L^−1^ rotenone-5 μmol·L^−1^ antimycin A for OCR or 10 mmol·L^−1^ glucose, 1 μmol·L^−1^ oligomycin and 50 mmol·L^−1^ 2-2-deoxyglucose (2-DG) for ECAR. Six replicates for each condition were used.

### ROS measurement

Total intracellular ROS levels in chondrocytes were detected by a Reactive Oxygen Species Assay Kit (Beyotime) based on the standard protocol. Mitochondrial ROS were detected by MitoSOX Red staining based on a standard protocol (Invitrogen^TM^, M36008).^82^ Chondrocytes were seeded on coverslips, washed with PBS and incubated with 5 μmol·L^−1^ MitoSOX for 10 min at 37 °C in the dark. Then, the cells were washed carefully with PBS and prepared for imaging under a confocal microscope.

### Ultrastructure visualization

For transmission electron microscopy (TEM) observation, cells were centrifuged to form pellets (~1 ×10^6^ cells for each pellet) and fixed with 2.5% glutaraldehyde at room temperature (RT) for 48 h. Then, the cells were rinsed 3 times in 1 mol·L^−1^ PBS buffer for 15 min each and postfixed in 1% OsO_4_ for 1 h. After 3 washes with PBS for 15 min each, the cells were dehydrated in a graded ethanol series (30%, 50%, 70%, 90%, 100%, 100%) and acetone (100%, 100%) for 20 min each and then embedded in Eponate 12 resin (Ted Pella). Sections were cut at a thickness of 50–80 nm and examined with Philips CM100 TEM at 80 kV. For confocal observation, chondrocytes were stained with 100 nmol·L^−1^ MitoTracker (Invitrogen) in serum-free medium in a dark cell incubator for 30 min. Then, they were fixed in 4% paraformaldehyde (PFA) solution for 20 min at RT and stained with DAPI (1:5 000, Beyotime) for 5 min. Photographs were taken by a Zeiss LSM 800 microscope.

### ATAC-seq

ATAC-seq was performed as previously described.^83^ Briefly, 5 ×10^4^ cells were harvested for each sample, centrifuged into pellets, and washed in 50 μL of cold PBS. Then, we gently resuspended the cell pellet in 50 μL of cold lysis buffer. The samples were centrifuged immediately at 500 × *g* for 10 min at 4 °C. After discarding the supernatant, we resuspended the samples in transposition reaction mix [25 μL of TD (2× reaction buffer), 2.5 μL of TDE1 (Nextera Tn5 Transposase), 22.5 μL of nuclease-free H_2_O] and incubated them at 37 °C for 30 min. We purified the transposed DNA with a Qiagen MinElute PCR Purification Kit. Next, the transposed DNA was amplified by PCR. The amplified library was purified with a Qiagen MinElute PCR Purification Kit. The quality of purified libraries was further assessed using a Bioanalyzer High Sensitivity DNA Analysis kit (Agilent). Libraries were sequenced on the Illumina HiSeq 2500 with 50 bp paired-end reads and aligned to the reference genome with Bowtie2. ATAC peaks were called using MACS2 with default parameters for each sample, and peaks in the same group were merged for downstream analysis. Chipseeker^84^ was utilized to annotate the peak locations, and group-specific peaks were identified by DiffBind^85^ with a threshold of FDR < 0.05 and log2 (fold change) > 2. A de novo motif search was performed using the HOMER package.^86,87^

### BTB06584 treatment

BTB06584 was purchased from MedChemExpress (MCE, HY-15877) and dissolved in dimethyl sulfoxide (DMSO, Sigma) at a 10 mmol·L^−1^ concentration as a stock reagent. Cells for BTB treatment were seeded and grown for 24 h until they were 40% confluent. Then, BTB was added to the freshly changed medium at a 2.5 μmol·L^−1^ concentration. DMSO (1/4 000) was used as a negative control. After 48 h, the BTB-containing medium was discarded, and the cells were washed with PBS and prepared for further tests.

### Bulk RNA sequencing

RNA-seq was modified based on a previous method.^88^ Briefly, RNA was extracted by TRIzol reagent (TaKaRa). Reverse transcription was conducted by SuperScript II reverse transcriptase (Invitrogen). Double strand cDNA was synthesized using the NEBNext mRNA second strand synthesis kit (NEB). A 3’ end-enriched sequencing library was constructed with a Nextera XT kit (Illumina) and sequenced on an Illumina X-Ten platform.

### Quantitative RT-PCR

PCR was performed using Brilliant SYBR Green QPCR Master Mix (TaKaRa) with a Light Cycler apparatus (ABI 7900HT). Primers: *Col2a1*: sense 5’CCACACCAAATTCCTGTTCA3’, antisense 5’ACTGGTAAGTGGGGCAAGAC3’; *Acan*: sense 5’AGGTCTGTGCCATCTGTGAG3’, antisense 5’CCGAGAAATGACACCTGCTA3’; antisense 5’CCGAGAAATGACACCTGCTA3’; *Sox9*: sense 5’GAGCCGGATCTGAAGAGGGA3’, antisense 5’GCTTGACGTGTGGCTTGTTC3’; *Sox6*: sense 5’CTGGCTGGGAACGACATGAT3’, antisense 5’TCGTCATAGGCTTCCATTTCATC3’; *Adamts5*: sense 5’CAGTGTGAAGCCAAAAATGGCTATC3’, antisense 5’TGCTGTACGGCCTGCATTCAGTCCC3’; *Mmp13*: sense 5’CTTTTCCTCCTGGACCAAACT3’, antisense 5’TCATGGGCAGCAACAATAAA3’.

### Immunofluorescence

Cells cultured on glass coverslips were fixed in 4% PFA for 20 min and then incubated in 0.3% Triton X-100, followed by incubation in blocking buffer (1% bovine serum albumin in PBS) for 30 min at RT. Afterward, samples were incubated with primary antibodies at 4 °C overnight and then with appropriate fluorescent probe-conjugated secondary antibodies for 2 h at RT. Cell nuclei were counterstained with DAPI. The primary antibodies were anti-COL2 (1:100, Novusbio, NB600-844), anti-aggrecan (1:100, Affinity DF7561), anti-MMP13 (1:100, Abcam, ab39012), anti-RBP4 (1:100, Abcam, ab109193), anti-SOD3 (1:50, Abcam, ab171738), anti-IFITM3 (1:100, Cell Signaling Technology, #59212; GeneTex, GTX115407), and Alexa Fluor 546 phalloidin (1:50, Invitrogen, A22283).

### Cell quality evaluation

Chondrocytes were seeded on coverslips and cultured for 24 h before examination. Briefly, the cells were fixed with 4% PFA and processed for immunofluorescence staining. Images were captured by a confocal microscope in a standard workflow (Zeizz LSM 880). Then, all images were transformed into TIFF format. The gray value averages and the cell/nucleus size in confocal images were calculated by Adobe Photoshop CC2015. Twenty cells per group were measured for each marker. The data were captured in a matrix (rows: relative intensity, round of detection from 1 to 20; column: group) and normalized into integers from 0 to 14 000. The data of P2 model cells from two batches were normalized into comparable values. The sample-to-sample distance heatmaps and PCA plots were drawn by DESeq2 from the R package.

In the micromass recovery test, chondrocytes were digested and suspended at 1 ×10^7^ cells per mL. Ten microliters of cell suspension was added to the dry surface at the center of a well in a 24-well plate. After incubation at 37 °C for 4 h, growth medium was gently added to the wells. The micromasses were cultured for 24 h and fixed for Alcian blue staining (Sigma).

### Statistical analysis

Statistical analysis was performed by GraphPad Prism 5 software. We used an unpaired *t* test for comparisons of two groups. To analyze data among three groups, we used one-way ANOVA to identify overall differences and Tukey’s test to compare all pairs of groups or Dunnett’s test to compare all groups *vs*. the control group. Data are shown as the mean ± SEM. *P < 0.05; **P < 0.01; ***P < 0.001.

## Supplementary information

Supplementary files

Table S1

Table S2

Table S3

Table S4

## Data Availability

The raw and processed data of scRNA-seq and ATAC-seq are available at the NCBI’s Gene Expression Omnibus (GEO) database with the accession ID: GSE193744, GSE193742, GSE193743.
