# Correction: A high-resolution route map reveals distinct stages of chondrocyte dedifferentiation for cartilage regeneration

**DOI:** 10.1038/s41413-022-00213-0

**Published:** 2022-05-18

**Authors:** Yishan Chen, Yeke Yu, Ya Wen, Juan Chen, Junxin Lin, Zixuan Sheng, Wenyan Zhou, Heng Sun, Chengrui An, Jiansong Chen, Weiliang Wu, Chong Teng, Wei Wei, Hongwei Ouyang

**Affiliations:** 1 Dr. Li Dak Sum & Yip Yio Chin Center for Stem Cells and Regenerative Medicine, and Department of Orthopedic Surgery of the Second Affiliated Hospital, Zhejiang University School of Medicine, Hangzhou, 310058 China; 2 Department of Sports Medicine, Zhejiang University School of Medicine, Hangzhou, 310058 China; 3 Zhejiang University-University of Edinburgh Institute, Zhejiang University School of Medicine, and Key Laboratory of Tissue Engineering and Regenerative Medicine of Zhejiang Province, Zhejiang University School of Medicine, Hangzhou, 310058 China; 4 Department of Oral Surgery, Ninth People’s Hospital, Shanghai Jiao Tong University School of Medicine, Shanghai, 200011 China; 5 Department of General Intensive Care Unit, The Second Affiliated Hospital of Zhejiang University School of Medicine, Hangzhou, 310009 China; 6 Department of Orthopedic Surgery, The Children’s Hospital, Zhejiang University School of Medicine, National Clinical Research Center for Child Health, Hangzhou, 310052 China; 7 Department of Orthopedic Surgery, The Fourth Affiliated Hospital, Zhejiang University School of Medicine, Yiwu, 322000 China; 8 China Orthopedic Regenerative Medicine Group (CORMed), Hangzhou, 310058 China

**Keywords:** Diseases, Energy metabolism

Correction to: *Bone Research* 10.1038/s41413-022-00209-w, published online 27 April 2022

Following the publication of the original article [1], it was noted that due to a typesetting error some elements in Fig. 5j were missing in the website version. The figure in the pdf version is correct.

The correct figure has been corrected in the website version.

