# Supplementary material for: A high-resolution route map reveals distinct stages of chondrocyte dedifferentiation for cartilage regeneration
Source: Bone Res. 2022 Apr 27;10:38. doi: 10.1038/s41413-022-00209-w (PMC9046296; doi:10.1038/s41413-022-00209-w)
Supplement: Supplementary file 1 — Supplementary files [file 41413_2022_209_MOESM1_ESM.pdf]

Supplementary files

Fig.S1. Establish a time-lapse chondrocyte dedifferentiation model. Related to Fig.1.

Fig.S2. TF coding genes along pseudo-time trajectory. Related to Fig.2.

Fig.S3. Representative features in MetC, when compared to EcmC and DegC. Related to Fig.3 and 4.

Fig.S4. Late dedifferentiated chondrocytes exhibit stress-associated chromatin remodeling. Related to Fig.4.

Fig.S5. Manipulating mitochondrial F1FoATPase efficiently ameliorates early dedifferentiation. Related to Fig.5.

Fig.S6. Manipulating mitochondrial F1FoATPase only partially ameliorate late dedifferentiation. Related to Fig.5.

Fig.S7. Immuno-staining of RNA-defined early and late dedifferentiation biomarkers in mouse chondrocyte dedifferentiation. Related to Fig.6.

Fig.S8. Early and late dedifferentiation biomarkers predict human chondrocyte plasticity. Related to Fig.6.

Fig.S9. Early and late dedifferentiation biomarkers predict the quality of heterogenous human chondrocytes. Related to Fig.6.

Table S1. List of cluster-specific markers. Related to Fig.1.

Table S2~3. List of dynamically regulated genes and TF coding genes along pseudo-time trajectory. Related to Fig. 2 and S2.

Table S4. Differentially expressed genes in negative control *vs* BTB-treated P2 chondrocytes, detected by bulk RNA-seq. Related to Fig. 5.

Table S5 Donor information of human chondrocytes. Related to Fig. 6, S8 and S9.

The raw and processed data of scRNA-seq and ATAC-seq are available at the NCBI's Gene Expression Omnibus (GEO) database with the accession ID: GSE193744, GSE193742, GSE193743.

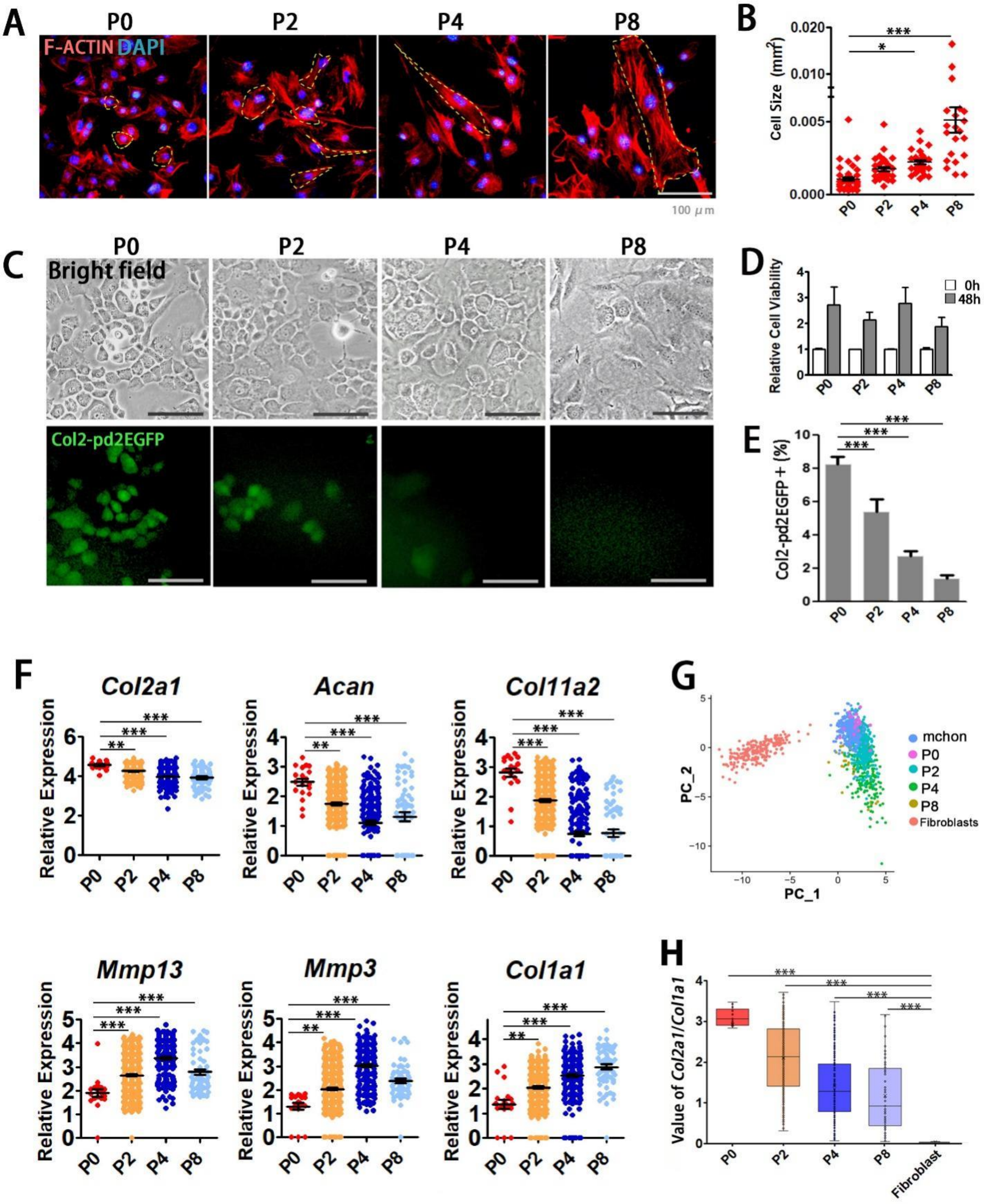

Fig. S1. Establish a time-lapse chondrocyte dedifferentiation model. Related to Fig.1.

(**A**) Phalloidin staining of filamentous actin (F-ACTIN) in P0-8 chondrocytes; scale bars: 100μm.

(**B**) Cell size quantitative analysis in P0-8 chondrocytes.

(**C**) Representative bright field and corresponding fluorescence images of P0-8 Col2-pd2EGFP reporter chondrocytes; scale bars: 100μm.

(**D**) Relative cell viability of P0-8 chondrocytes.

(**E**) Percentage of Col2-pd2EGFP^{+} cells in P0-8 chondrocytes.

(**F**) Relative expression of representative dedifferentiation markers in P0-8 chondrocytes.

(**G**) Principle component analysis (PCA) plotting showing the comparison of P0-P8 cells in this study with mouse primary chondrocytes and fibroblasts from another data resource (GSE118236).
(**H**) Ratio of normalized *Col2a1*expression to normalized *Col1a1* expression in P0-8 chondrocytes, compared to murine fibroblast scRNA-seq data (GSE118236).
All data were mean ± SEM. * p<0.05, ** p<0.01, *** p<0.001.

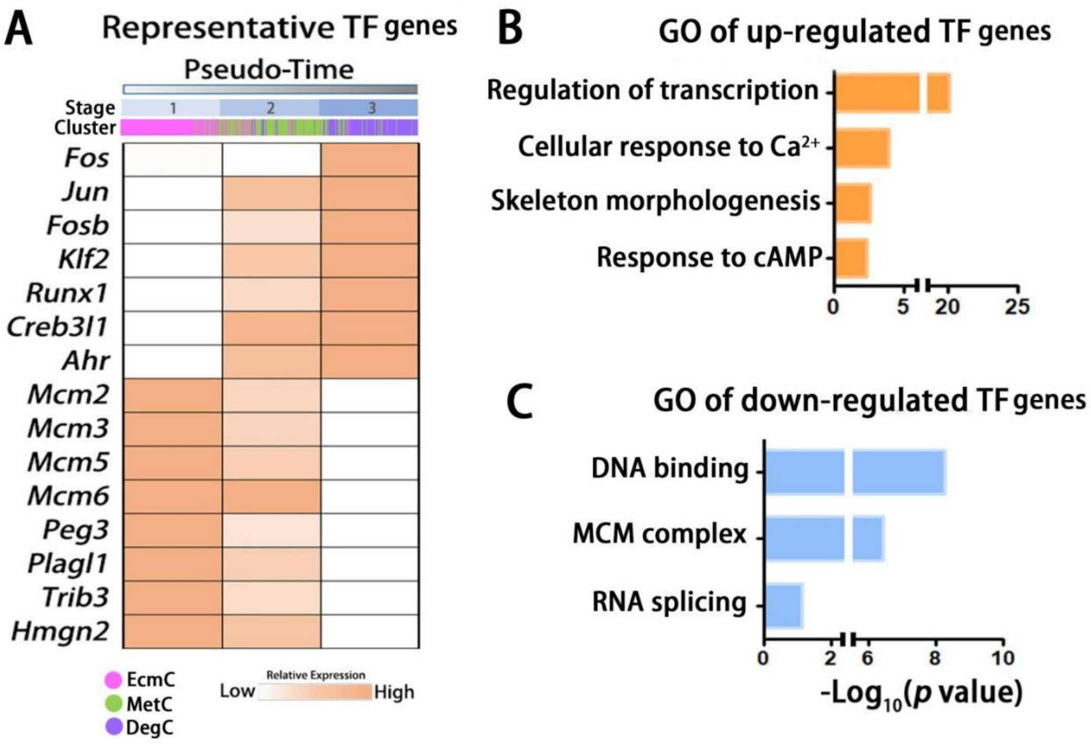

Fig. S2. Demonstrate TF coding genes along pseudo-time trajectory. Related to Fig.2.

(**A**) Heatmap of representative TF coding genes expressed along the pseudo-time trajectory.

(**B**)~(**C**) Gene ontology (GO) analysis of up- and down-regulated TF coding genes.

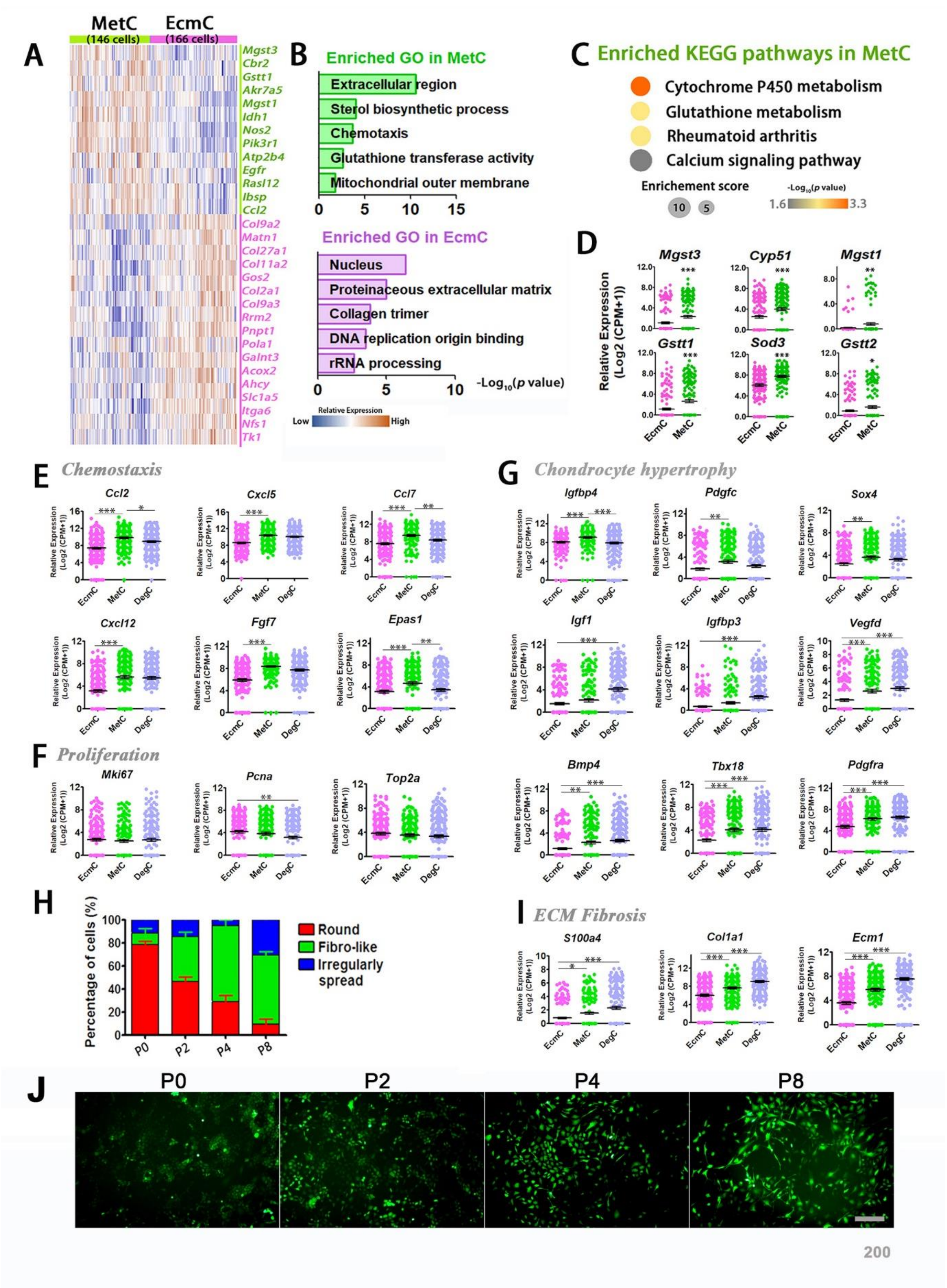

Fig. S3. Representative features in MetC, when compared to EcmC and DegC. Related to Fig. 3 and 4.

(**A**)~(**B**) Heatmap of differentially expressed genes in MetC vs EcmC, and enriched GO terms.
(**C**)~(**D**) Enriched KEGG pathways and example genes in MetC.
(**E**) Representative genes of chemotaxis in EcmC, MetC and DegC.
(**F**) Representative genes of proliferation in EcmC, MetC and DegC.
(**G**) Representative genes of tissue development and chondrocyte hypertrophy highly expressed in MetC and DegC.
(**H**) Cell shape quantitative analysis in P0-8 chondrocytes.

(**I**) Representative genes of tissue development, chondrocyte hypertrophy and fibrosis highly expressed in DegC.

(**J**) Representative images of the ROS detection in P0-8 chondrocytes using a Reactive Oxygen Species Assay Kit (Beyotime). The fluorescence intensity represents the relative level of total intracellular ROS in cells.

All data were mean± SEM. * p<0.05, ** p<0.01, *** p<0.001.

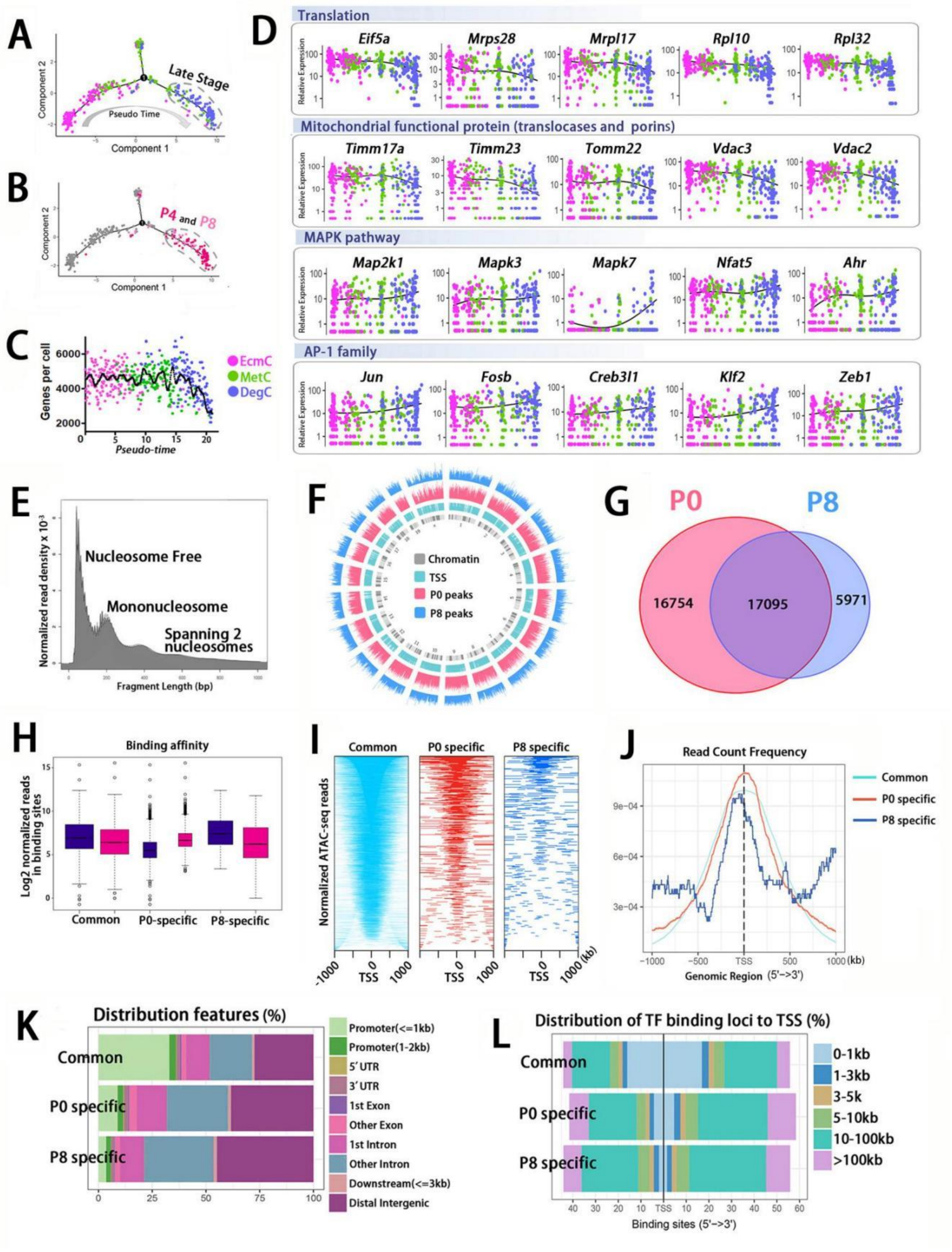

Fig.S4. Late dedifferentiated chondrocytes exhibit stress-associated chromatin remodeling. Related to Fig.4.

(**A**) Pseudo-time trajectory profiles of cells marked in DegC as late dedifferentiated cluster.

(**B**) Pseudo-time trajectory profiles of cells marked in P4 and 8 as late dedifferentiated passages.

(**C**) Genes expressed per cell in EcmC, MetC and DegC.

(**D**) Expression of typical genes responsible for translation, mitochondrion function proteins, MAPK pathway, AP-1 family factors along the pseudo-temporal axis.

(**E**) Density of ATAC-seq fragments patterns of mono- and di-nucleosomes.

(**F**) Circos plot showing genome wide chromatin accessibility in P0 and P8 chondrocytes.

(**G**) Venn plot showing the quantity of common, P0 and P8 specific peaks, identified by Diffbind.

(**H**) Box plots of read distributions for significantly differentially bound sites in P0 and P8 chondrocytes.

(**I**) Heatmap showing density of annotated ATAC-seq reads 1kb up and downstream of transcription start sites (TSS).
(**J**) Frequency of ATAC-seq reads of common, P0 and P8 specific peaks
(**K**) Distribution features of common, P0 and P8 specific peaks.
(**L**) Distribution of common, P0 and P8 specific TF binding loci.

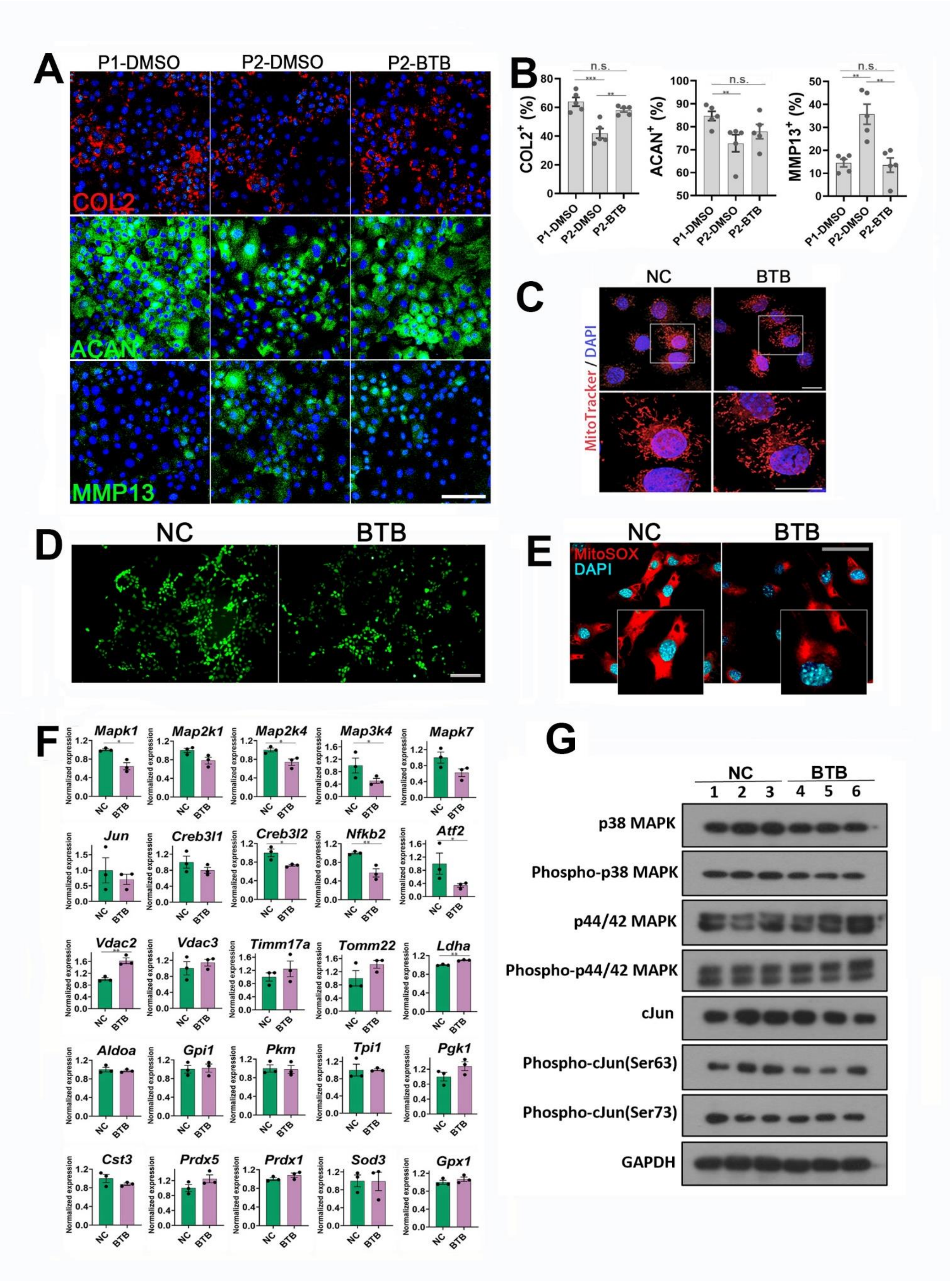

Fig.S5. Manipulating mitochondrial F1FoATPase efficiently ameliorates early dedifferentiation. Related to Fig.5.

(**A**)~(**B**) Immunostaining images and quantitative data of COL2, ACAN and MMP13 in BTB-untreated (DMSO as negative control) P1, P2 and treated P2 chondrocytes; scale bars: 100μm.

(**C**) Representative images of MitoTracker-stained mitochondria in NC and BTB-treated P2 chondrocytes; scale bars: 20 μm.

(**D**) Representative images of the ROS detection in BTB-untreated (negative control, NC) and treated P2 chondrocytes using a Reactive Oxygen Species Assay Kit (Beyotime); scale bars: 200μm. The fluorescence intensity represents the relative level of total intracellular ROS in cells.

(**E**) Representative images of relative mitochondrial ROS production in NC and treated P2 chondrocytes, detected by MitoSOX Red staining; scale bars: 50μm.

(**F**) Typical genes of MAPK pathway, mitochondrion-to-nuclear effectors, and mitochondrion function proteins in BTB-treated P2 chondrocytes *vs* NC, detected by bulk RNA-seq.

(**G**) The protein expression level and phosphorylation status of representative proteins in MAPK pathway and the downstream targets in NC and BTB-treated P2 chondrocytes, detected by Western blot.

The final concentration of BTB was 2.5μM. "NC" (negative control) in this figure represents the treatment by 0.025% dimethyl sulfoxide (DMSO), the solvent of BTB.

All data were mean± SEM. * p<0.05, ** p<0.01, *** p<0.001.

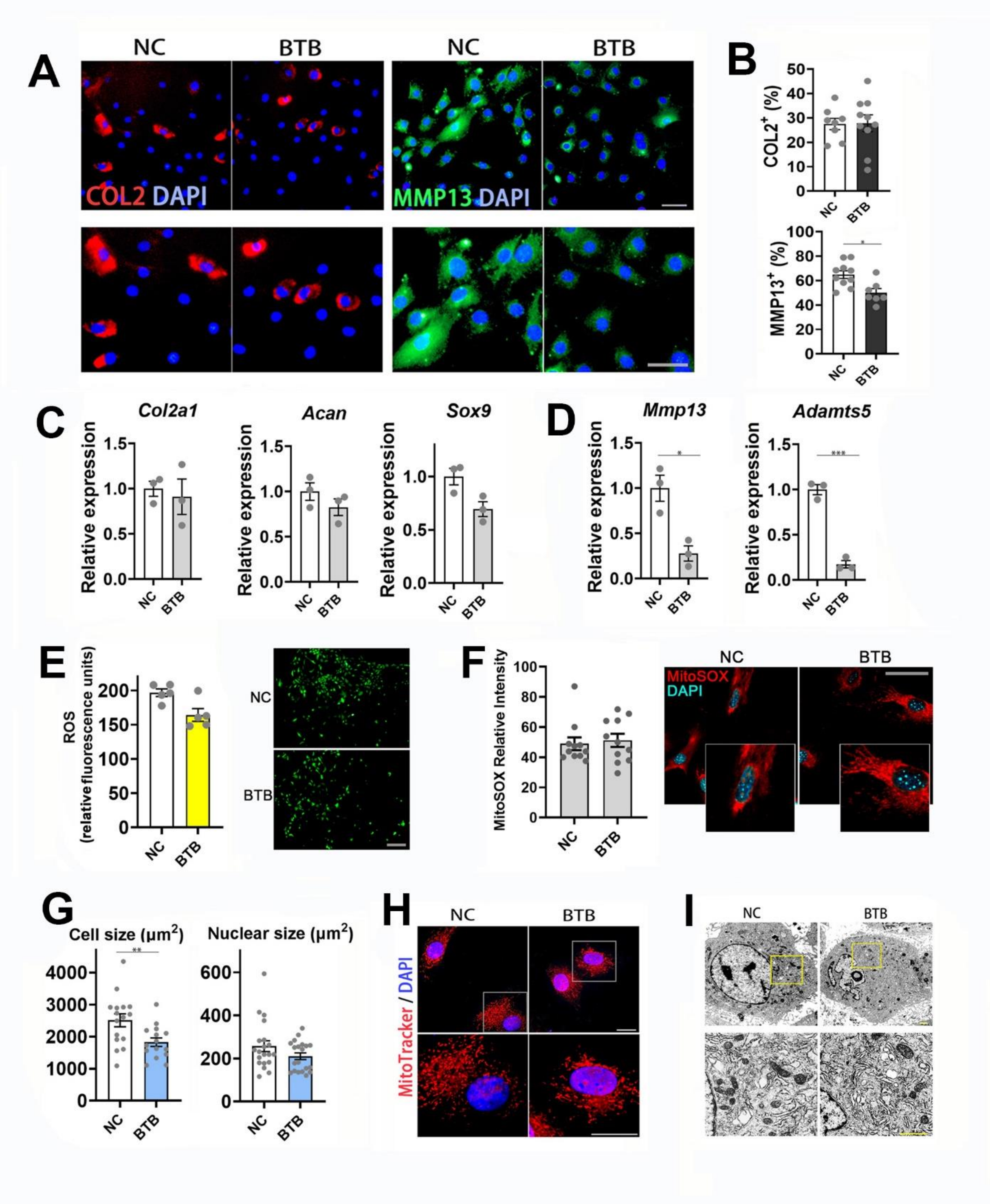

Fig.S6. Manipulating mitochondrial F1FOATPase only partially ameliorate late dedifferentiation. Related to Fig.5.

(**A**)~(**B**) Immunostaining images and quantitative data of COL2 and MMP13 in BTB-untreated (negative control, NC) and treated P4 chondrocytes; scale bars: 50μm.

(**C**)~(**D**) qPCR detection of representative dedifferentiation genes in NC and BTB-treated P4 chondrocytes.

(**E**) Relative total intracellular ROS production in NC and BTB-treated P4 chondrocytes; representative images and quantitative analysis; scale bars: 200μm.

(**F**) Relative mitochondrial ROS production in P0-8 chondrocytes and the quantitative data, detected by MitoSOX Red staining; scale bars: 50μm.

(**G**) Quantitative analysis of cellular and nuclear size of NC and BTB-treated P4 chondrocytes.

(**H**) Representative images of MitoTracker-stained mitochondria in NC and BTB-treated P4 chondrocytes; scale bars: 20 μm.

(**I**) Representative images of mitochondria in NC and BTB-treated P4 chondrocytes, by transmission electron microscopy; scale bars: 1μm.

The final concentration of BTB was 2.5μM. "NC" (negative control) in this figure represents the treatment by 0.025% dimethyl sulfoxide (DMSO), the solvent of BTB.

All data were mean± SEM. * p<0.05, ** p<0.01, *** p<0.001.

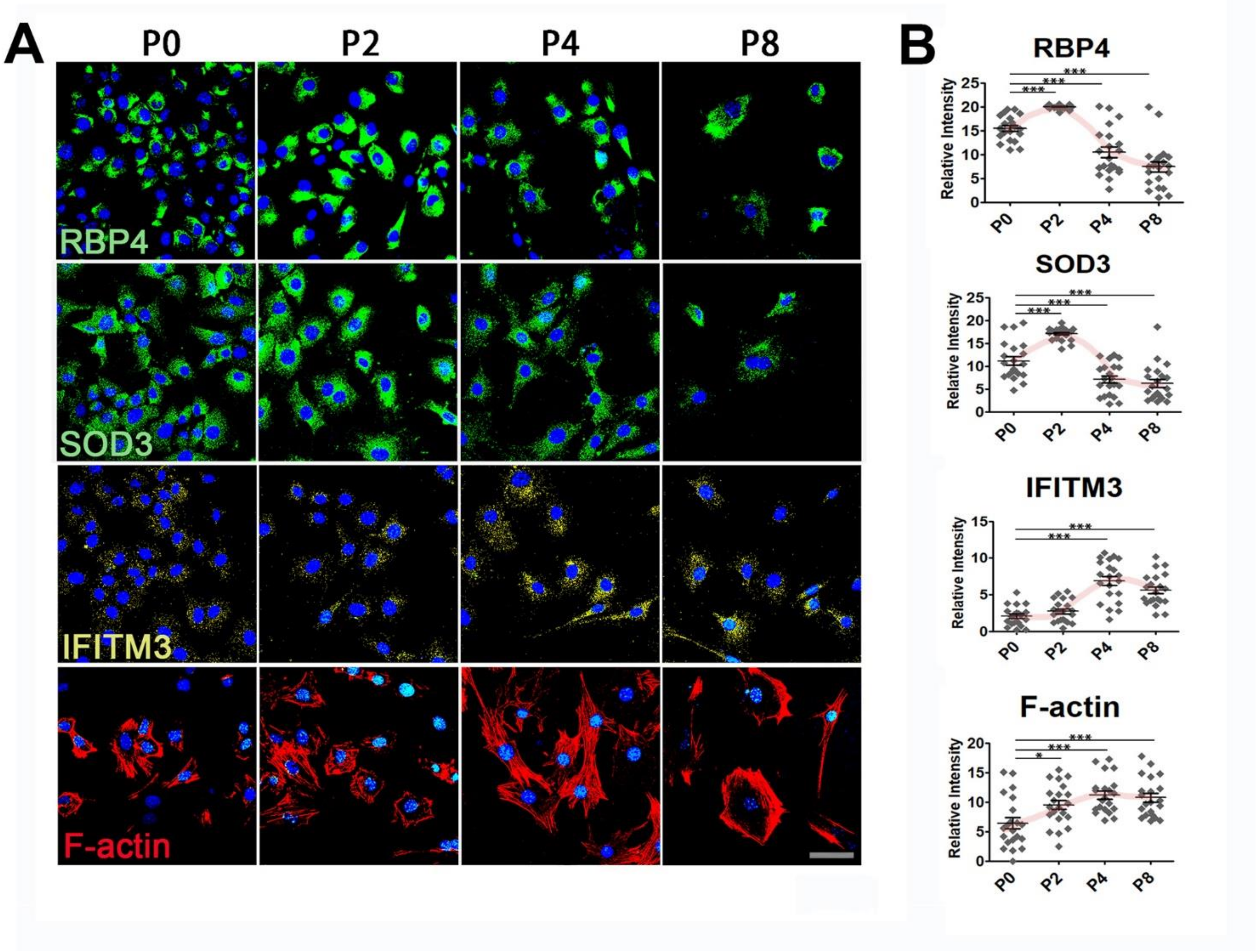

Fig.S7. The validation on the protein level of RNA-seq defined early and late dedifferentiation biomarkers in mouse chondrocyte dedifferentiation. Related to Fig.6.

(**A**) Representative images of immuno-stained RBP4, SOD3, IFITM3 and F-actin in mouse P0-8 chondrocytes; scale bars: 50μm.

(**B**) Quantitative data of immuno-stained RBP4, SOD3, IFITM3 and F-actin relative intensity.
All data were mean± SEM. * p<0.05, ** p<0.01, *** p<0.001.

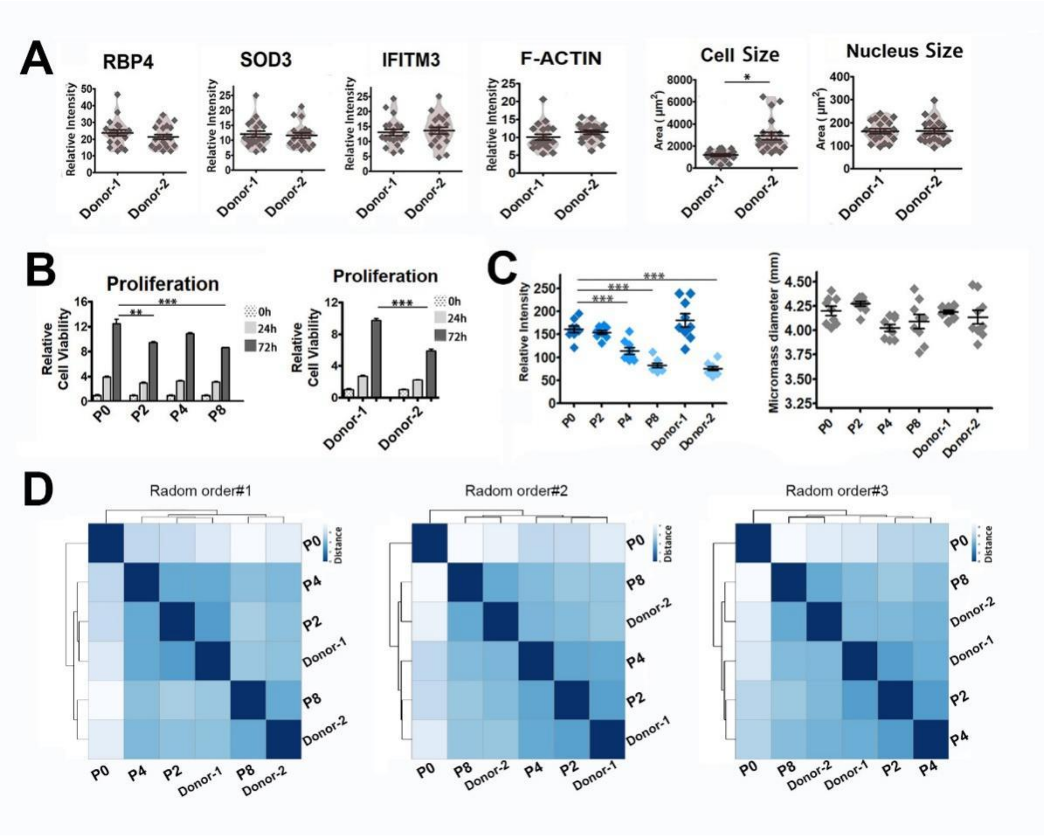

Fig.S8. Early and late dedifferentiation biomarkers predict human chondrocyte plasticity. Related to Fig.6.

(**A**) Quantitative data of immuno-stained RBP4, SOD3, IFITM3, F-actin relative intensity, cell size and nucleus size in human articular chondrocytes from Donor-1 and 2.

(**B**) Relative intensity of Alcian blue staining and diameter of micromasses formed by model human chondrocytes (P0-8) and articular chondrocytes from Donor-1 and 2.

(**C**) Relative cell viability of model human chondrocytes (P0-8) and articular chondrocytes from Donor-1 and 2.

(**D**) Sample-to-sample distance heatmaps showing the similarity of Donor-1 and 2 chondrocytes with human chondrocyte dedifferentiation model (P0-8). The values in some parameters were randomly chosen to be reordered. For example, the values of RBP4_intensity_round1, RBP4_intensity_round2…round20 of P4, were reordered. Random order # 1~3: the sample distances were calculated from 3 matrix in which some values were in different random orders. This is to confirm the test order will not significantly alter the results of sample correlation.

All data were mean± SEM. * p<0.05, ** p<0.01, *** p<0.001.

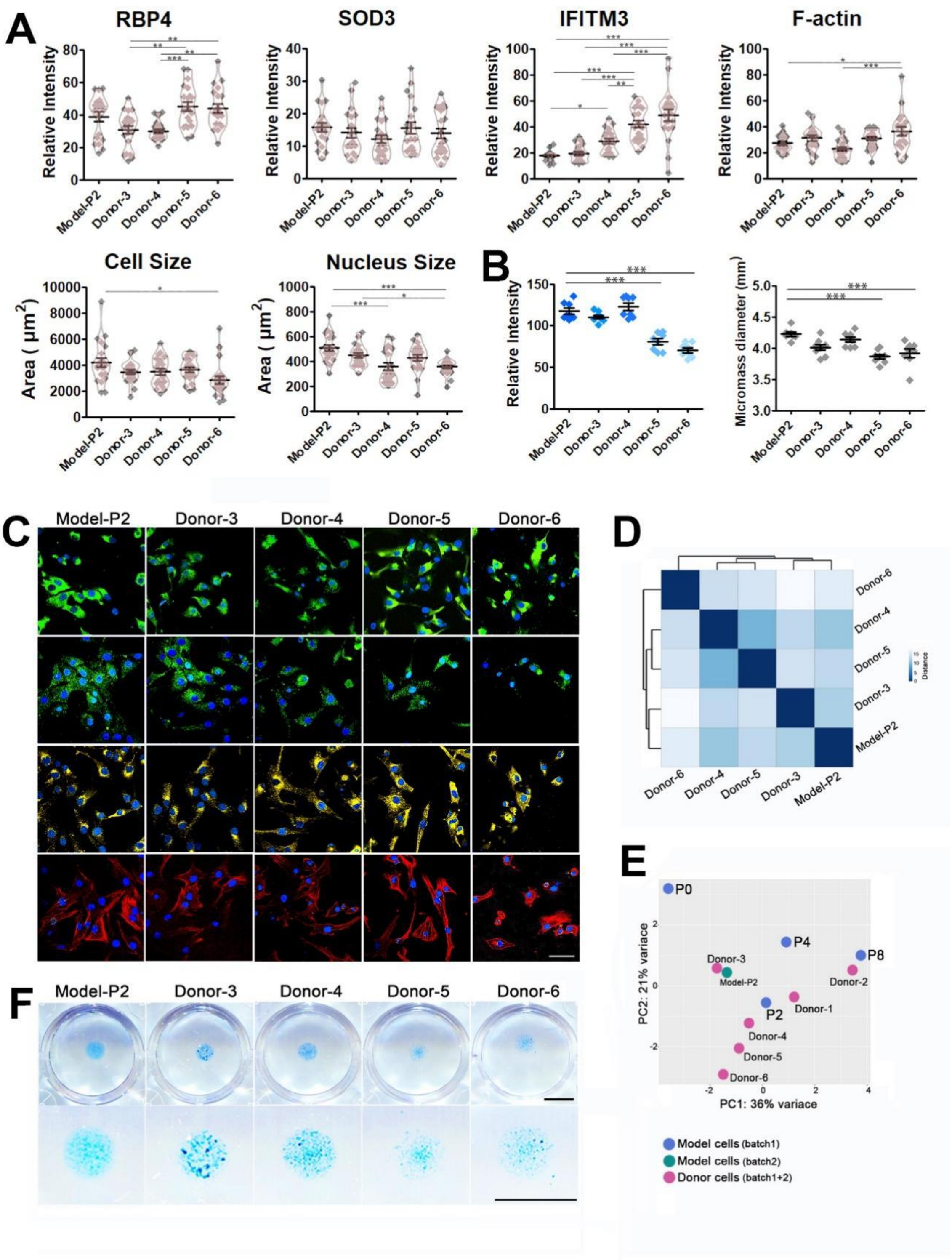

Fig.S9. Early and late dedifferentiation biomarkers predict the quality of heterogenous human chondrocytes. Related to Fig.6.

(**A**) Quantitative data of immuno-stained RBP4, SOD3, IFITM3, F-actin relative intensity, cell size and nucleus size in model chondrocytes (P2), and articular chondrocytes from Donor-3,4,5 and 6.

(**B**) Relative intensity of Alcian blue staining and diameter of micromasses formed by odel chondrocytes (P2), and articular chondrocytes from Donor-3,4,5 and 6.

(**C**) Representative images of immuno-stained RBP4, SOD3, IFITM3, F-actin in model chondrocytes (P2), and articular chondrocytes from Donor-3,4,5 and 6; scale bars: 50μm.

(**D**) Sample-to-sample distance heatmap of model chondrocytes (P2), and articular chondrocytes from Donor-3,4,5 and 6.

(**E**) Principle component analysis (PCA) plotting shows the relationship of all evaluated chondrocytes. Model cells (blue dots): P0-8 healthy chondrocytes in batch#1; model cells (green dots): P2 healthy chondrocytes in batch#2; donor cells (violet dots): chondrocytes from different donors for measurement.

(**F**) Alcian blue–stained micromasses formed by model chondrocytes (P2), and articular chondrocytes from Donor-3,4,5 and 6; scale bars: 5 mm.

All data were mean± SEM. * $p<0.05$, ** $p<0.01$, *** $p<0.001$.

Table S5. Donor information of human chondrocytes

Group	Passage	Age	Sex	Condition	Tissue
Model	P0~8	9 m	Female	Polydactyly	Polydactyly-derived cartilage
Model	P0~8	11 m	Female	Polydactyly	Polydactyly-derived cartilage
Model	P2	15 m	Male	Polydactyly	Polydactyly-derived cartilage
Model	P2	12 m	Male	Polydactyly	Polydactyly-derived cartilage
Model	P2	12 m	Female	Polydactyly	Polydactyly-derived cartilage
Model	P2	24 m	Female	Polydactyly	Polydactyly-derived cartilage
Donor-1	P2	17 w	Female	Abortion	Knee uncalcified cartilage
Donor-2	P2	55 y	Female	OA	Undamaged articular cartilage
Donor-3	P2	54 y	Male	Trauma	Normal articular cartilage
Donor-4	P2	75 y	Female	OA	Undamaged articular cartilage
Donor-5	P2	72 y	Male	OA	Undamaged articular cartilage
Donor-6	P2	65 y	Male	OA	Undamaged articular cartilage
